# Integrative multi-omics Mendelian randomization and functional validation identifies RNASET2 as a novel therapeutic target for autoimmune thyroiditis

**DOI:** 10.3389/fendo.2026.1715937

**Published:** 2026-02-02

**Authors:** Bo Jiang, Yanxue Wang, Cheng Qu, Chen Zhang, Chaoyu Jiang, Lei Su, Wenxian Guan, Yuqian Luo

**Affiliations:** 1 Department of General Surgery, Nanjing Drum Tower Hospital, Clinical College of Nanjing Medical University, Nanjing, China; 2 Department of Thyroid Surgery, Nanjing Drum Tower Hospital, The Affiliated Hospital of Nanjing University Medical School, Nanjing, China; 3 Department of General Surgery, Nanjing Drum Tower Hospital, The Affiliated Hospital of Nanjing University Medical School, Nanjing, China; 4 Clinical Medicine Research Center, Nanjing Drum Tower Hospital, The Affiliated Hospital of Nanjing University Medical School, Nanjing, China

**Keywords:** autoimmune thyroiditis, Hashimoto’s thyroiditis, Mendelian randomization, multi-omics, RNASET2, thyrocyte spheroids

## Abstract

**Objective:**

Autoimmune thyroiditis (AIT), a prevalent autoimmune disorder that frequently leads to hypothyroidism. A critical unmet need exists for disease-modifying therapies that target its underlying pathogenesis. This study aimed to identify and validate novel therapeutic targets for AIT.

**Methods:**

We employed an integrative genomics approach, combining genome-wide association studies (GWAS) with molecular quantitative trait loci (QTL) analyses, including expression (eQTL), protein (pQTL), and DNA methylation QTL (mQTL), across two independent AIT cohorts for discovery and replication. We performed two-sample bidirectional Mendelian randomization (MR) with sensitivity analyses, followed by summary-data-based MR (SMR) and heterogeneity in dependent instruments (HEIDI) tests. Top candidates were further evaluated via phenome-wide association study (PheWAS) and computational drug screening. Guided by these findings, we quantified plasma levels of the top-priority candidate, Ribonuclease T2 (RNASET2), via ELISA in AIT patients and non-AIT controls. To functionally validate its therapeutic potential, we developed a novel three-dimension (3D) inflammatory thyrocyte spheroid model and evaluated potential therapeutic effects of recombinant RNASET2. Loss-of-function (small interfering RNA-mediated knockdown) and gain-of-function (recombinant protein RNASET2 rescue) experiments further supported RNASET2 as a therapeutic target.

**Results:**

Multi-omics integration consistently nominated RNASET2 as a causal protective factor against AIT. Signals of pQTL and eQTL for RNASET2 were associated with decreased AIT risk, while three mQTLs were correlated with increased risk. PheWAS indicated minimal pleiotropic effects, supporting its therapeutic suitability. Computational drug screening nominated genistein, a soy isoflavone known to upregulate RNASET2 expression, as a repurposing candidate. Empirically, plasma RNASET2 levels were moderately elevated in AIT patients, potentially reflecting a compensatory anti-inflammatory response. Crucially, recombinant RNASET2 effectively mitigated inflammation and apoptosis in the thyrocyte spheroid model, confirming its functional protective role. Consistently, RNASET2 knockdown heightened susceptibility to inflammatory cell death and cytokine expression, a phenotype reversed by recombinant RNASET2 supplementation.

**Conclusions:**

By integrating large-scale genomic analyses with functional validation, our study establishes RNASET2 as a promising therapeutic target for AIT. RNASET2 augmentation represents a potential disease-modifying strategy, providing a translational bridge from genetic discovery to clinical application.

## 1 Introduction

Autoimmune thyroiditis (AIT), also known as Hashimoto’s thyroiditis (HT), is a chronic thyroid autoimmune disorder characterized by immune-mediated destruction of the thyroid gland, often leading to hypothyroidism. Its common clinical manifestations include fatigue, weight gain, cold intolerance, depression, and goiter formation (1). Current treatment primarily relies on lifelong thyroid hormone replacement therapy (e.g., levothyroxine) to manage hypothyroidism (1); however, this approach fails to address the underlying autoimmune pathogenesis. The major challenge in curing AIT stem from its complex etiology, involving genetic predisposition, environmental triggers, and dysregulated immune responses. To date no disease-modifying therapies are available to halt or reverse the autoimmune process, leaving patients at risk of progressive thyroid damage and potential systemic complications. Given the limitations of existing treatment and the growing prevalence of AIT, developing targeted therapies and novel medications that directly modulate thyroid autoimmunity represents a critical unmet medical need.

Effective drug development relies on the accurate identification of therapeutic targets and rigorous validation of their disease-modifying on a disease. However, conventional drug discovery approaches face significant challenges including high costs, lengthy timelines, and substantial failure rates. To overcome these limitations, innovative strategies integrating genomic data are essential for accelerating the process of therapeutic target identification (2). In recent years, the combined analysis of genome-wide association studies (GWAS) with molecular quantitative trait loci (QTL) data, such as expression QTLs (eQTLs), DNA methylation QTLs (mQTLs), and protein QTLs (pQTLs), provides a powerful framework for establishing causal relationships between genetic variants and disease-associated molecules (3, 4). This integrative approach enhances the discovery of biologically relevant drug targets while improving the efficiency of the drug development pipeline.

Mendelian randomization (MR) analysis serves as a powerful analytical approach to assess causal relationships between exposures and outcomes by leveraging genetic variants as instrumental variables (IVs), effectively mimicking a randomized controlled trial using observational genetic data (5). This method provides a robust alternative to traditional drug testing by circumventing confounding factors and reverse causation inherent in conventional observational studies (5). Notably, MR analysis has been successfully applied to discover drug targets in several autoimmune disorders (6–9), offering novel insights into their pathogenesis and potential therapeutic targets.

Following computational target identification, *in vitro* validation represents a crucial next step. The transition from traditional two-dimensional (2D) monolayer cultures to three-dimensional (3D) cell spheroids represents a significant advancement, primarily due to their superior ability to mimic the complex *in vivo* microenvironment. Unlike flat monolayers, spheroids recapitulate critical physiological characteristics such as cell-cell and cell-matrix interactions, gradient formation for nutrients, oxygen, and drug penetration, and the development of proliferative and quiescent cell zones (10). This architecture leads to more physiologically relevant gene expression, metabolism, and response to therapeutic agents, thereby providing a more accurate and predictive model for drug efficacy, toxicity, and penetration studies. It is precisely this enhanced pathophysiological relevance that makes 3D spheroids an ideal system for validating the anti-inflammatory drug candidates identified through large-scale data analysis. By better modeling the tissue-like environment where inflammation occurs, 3D models allow for a more faithful assessment of a compound’s ability to modulate inflammatory pathways, effectively bridge the gap between computational prediction and biological reality. Consequently, drug validation using 3D spheroids reduces the high attrition rates in drug development by yielding data that is more translatable to clinical outcomes, ultimately leading to the identification of more effective and safer candidates.

In this study, we aimed to identify novel therapeutic targets for AIT by selecting instrumental variables associated with eQTLs, mQTLs, and pQTLs to investigate their causal effects on gene expression, epigenetic modifications, and protein levels in AIT. To enhance the robustness of our findings, we validated the results using two independent GWAS datasets of AIT and employed a two-sample MR analysis as our primary analytical approach, complemented with summary-data-based MR (SMR) analysis and heterogeneity in dependent instruments (HEIDI) tests. Our integrative genomic and epigenomic analyses establish RNASET2 as a causal protective factor in AIT, positioning it as a promising therapeutic target. Also, we conducted a pharmacological evaluation to assess its translational potential of our findings for AIT treatment. Furthermore, we quantified the plasma RNASET2 levels in AIT patients and non-ATI controls and pharmacologically evaluated the effects of RNASET2 in a novel human inflammatory thyrocyte spheroid model, providing functional validation of its therapeutic utility in a disease-mimicking tissue-like setting.

## 2 Materials and methods

### 2.1 Ethical approval

The large-data used in our analysis were publicly available and had been approved by the respective institutional review boards of the original studies. Human samples used in this study were obtained with written consent, according to the guidelines approved by the Ethical Committee of the institutional review boards (IRB) of Nanjing Drum Tower Hospital, Nanjing, China (IRB Review Approval Number: 2022-096-02).

### 2.2 Study design of a large-data analysis

**Figure 1:**
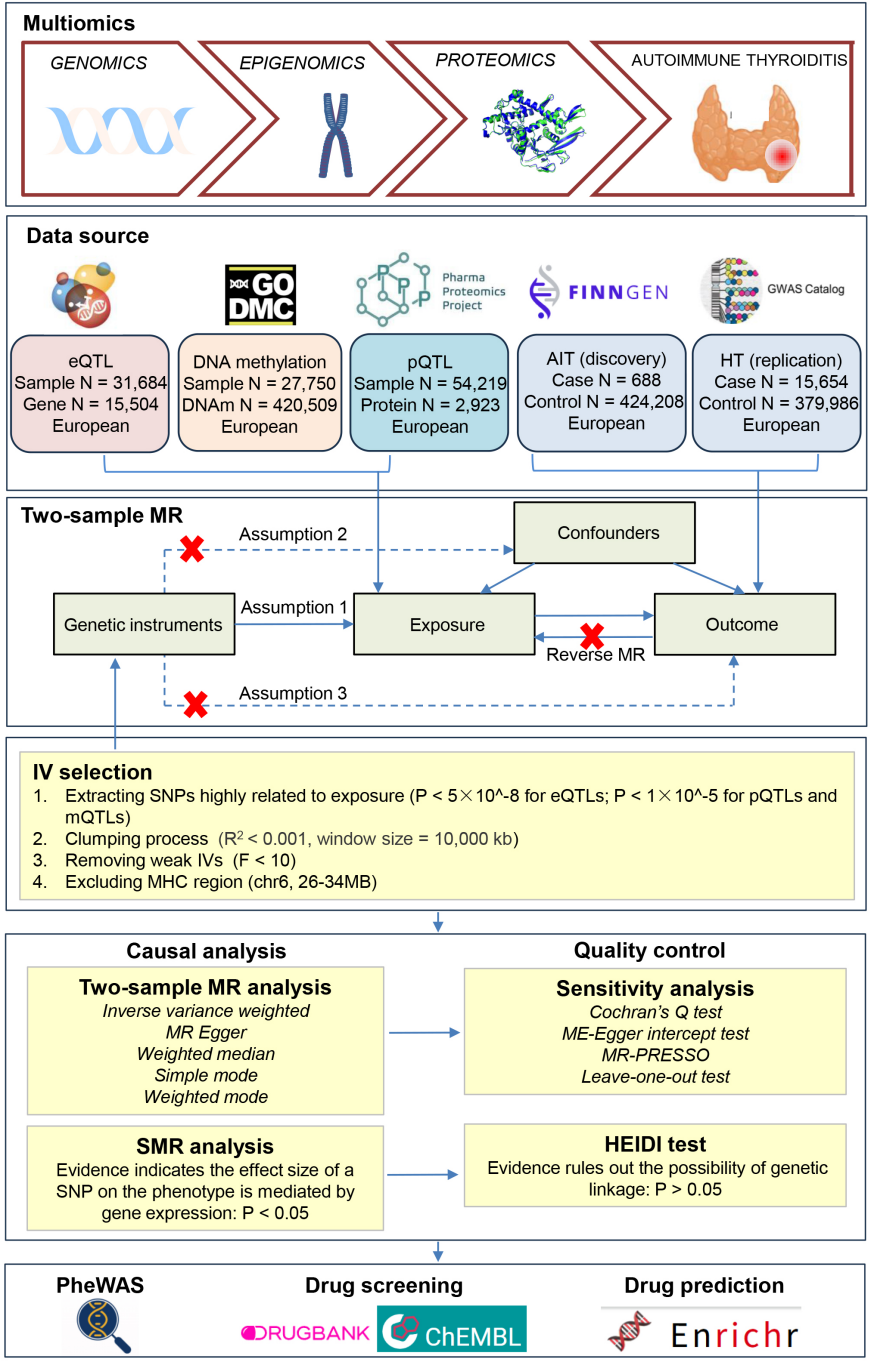
The workflow of large-data analysis is illustrated in the flow chart. We integrated GWAS data for molecular QTLs (cis-eQTLs, pQTLs, and mQTLs) with two independent AIT GWAS datasets (discovery and replication cohorts) to investigate causal relationships. After rigorous IV selection, we conducted two-sample MR analyses to assess causal effects of eQTLs and pQTLs on AIT risk, with sensitivity analyses for reverse causation, horizontal pleiotropy and heterogeneity, and with cross-validation of directional effects between cohorts. Significant findings underwent additional validation through SMR and HEIDI test at the gene expression level, followed by mQTL-specific MR analysis to evaluate DNA methylation effects of the identified gene on AIT. Finally, we performed phenome-wide association study (PheWAS), drug screening, and computational drug prediction for the identified target.

Our large-data analysis (**Figure 1**) integrated GWAS data for molecular QTLs (cis-eQTLs, pQTLs, and mQTLs) with two independent AIT GWAS datasets (discovery and replication cohorts) to investigate causal relationships. After rigorous IV selection, we conducted two-sample MR analyses to assess causal effects of gene expression and protein levels on AIT risk, with sensitivity analyses for reverse causation, horizontal pleiotropy, and heterogeneity, and with cross-validation of directional effects between cohorts. Significant findings underwent additional validation through SMR and HEIDI test at the gene expression level, followed by mQTL-specific MR analysis to evaluate DNA methylation effects of the identified gene on AIT. Finally, we performed phenome-wide association study (PheWAS) and computational drug prediction for the identified target, with detailed methodological specifications provided in subsequent sections.

### 2.3 Data sources

We obtained GWAS data for AIT from two independent sources: 1) the FinnGen consortium’s latest release (R12), comprising 688 cases and 424,208 controls (accession: finngen_R12_E4_THYROIDITAUTOIM), and 2) the IEU Open GWAS project with 15,654 cases and 379,986 controls (accession: ebi-a-GCST90018855) (11). For molecular QTLs, we utilized: (i) cis-eQTL data from the eQTLGen consortium (https://eqtlgen.org/) (31,684 blood samples; 16,989 genes) (12), (ii) pQTL data from UKB-PPP (http://ukb-ppp.gwas.eu) (54,219 participants; 2,923 plasma proteins) (13), and (iii) mQTL data from GoDMC (http://mqtldb.godmc.org.uk/) (27,750 blood samples; 420,509 DNA methylation sites) (14). Additionally, we referred druggable gene information from established pharmacogenomic resources (15) to facilitate therapeutic target identification. All data sources are publicly available through their respective consortium portals.

### 2.4 Selection of IVs

To ensure valid causal inference using genetic variants as IVs, we adhered to three core MR assumptions: 1) strong association between genetic variants and exposure (relevance assumption), 2) absence of confounding between genetic variants and outcome (independence assumption), and 3) exclusion of pleiotropic pathways not mediated by the exposure (exclusion restriction assumption). Our IV selection protocol incorporated the following stringent criteria: First, we identified potential IVs through genome-wide screening of molecular QTLs, applying locus-specific significance thresholds (P < 5×10–^8^ for eQTLs; P < 1×10–^5^ for pQTLs/mQTLs). Second, we performed linkage disequilibrium (LD)-based clumping using European samples from the 1000 Genomes Project as reference, retaining only independent variants (R^2^ < 0.001 within 10,000 kb windows) with the strongest association signals (16). Third, we evaluated instrument strength using the F-statistic [(N-K-1)/K × R^2^/(1-R^2^)], excluding variants with F < 10 to mitigate weak instrument bias (17). Finally, we systematically removed confounding variants: those in the MHC region (Chr6:26–34 Mb) (18), to ensure the validity of our causal estimates.

### 2.5 Two-sample MR analysis

We conducted a comprehensive two-sample MR analysis to investigate potential causal relationships between molecular QTLs and AIT. Our primary analytical approach utilized the inverse variance weighted (IVW) method, which combines Wald ratio estimates from individual SNPs through meta-analysis to provide robust causal effect estimates (19). The analysis protocol consisted of three key steps: (1) harmonization of SNP alleles across datasets to ensure proper matching, (2) calculation of Wald ratio estimates for each individual SNP, and (3) meta-analysis integration using the IVW approach. This method offers enhanced precision by leveraging multiple genetic variants simultaneously while maintaining unbiased estimates in the absence of horizontal pleiotropy. Effect sizes were expressed as odds ratios (ORs) derived from exponentiated β coefficients, accompanied by 95% confidence intervals (CIs), with statistical significance defined as P < 0.05.

To ensure the robustness of our findings, we implemented multiple validation strategies: (1) sensitivity analyses using MR-Egger regression (which corrects for pleiotropic bias through linear regression modeling) (20), weighted median estimator (providing reliable estimates under sample bias) (21), simple mode and weighted mode approaches (reducing confounding through randomization techniques) (22); (2) stringent exclusion of potential reverse causation by retaining only SNPs with greater variance explained in exposure than outcome; and (3) preservation of data integrity by abstaining from missing value imputation. All analyses were performed using the “TwoSampleMR” package within the R 4.3.2 statistical environment (available at https://cran.r-project.org/bin/windows/base/).

### 2.6 Sensitivity analysis

Heterogeneity among the IVs was assessed using Cochran’s Q test, calculated within the IVW framework. Cochran’s Q test is a widely used method to evaluate heterogeneity across different IVs in a study (23). The P-value obtained from this test is critical in determining the presence or absence of significant heterogeneity. P < 0.05 indicates significant heterogeneity among the IVs. To address potential horizontal pleiotropy, the MR-Egger intercept test was employed (20). The significance of the intercept term in the MR-Egger regression suggests the presence of horizontal pleiotropy. Additionally, the MR pleiotropy residual sum and outlier (MR-PRESSO) method was applied to identify and exclude outliers with horizontal pleiotropic effects that could significantly bias the MR estimates (24). To visually assess the robustness of the results, scatter plots and funnel plots were generated. Scatter plots confirmed that the findings were not influenced by outliers, while funnel plots demonstrated the consistency and absence of heterogeneity in the correlations. Finally, the “leave-one-out” method was used to evaluate the impact of individual SNPs on the MR estimates. This approach involved iteratively excluding one SNP at a time to determine whether the removal of any single SNP substantially altered the causal estimates, thereby ensuring the stability and reliability of the results.

### 2.7 SMR analysis and HEIDI test

The SMR and HEIDI approach offers a distinct advantage over most other integrative methods for analyzing GWAS and eQTL data by enabling differentiation between pleiotropic and linkage models (25). We employed SMR analysis as a supplementary approach to validate causal relationships between AIT and gene expression. The HEIDI test was specifically applied to rule out the possibility of genetic linkage (26). Following established criteria, we defined significant SMR associations as those with P < 0.05, while HEIDI results with P > 0.05 indicated associations mediated by shared genetic variants rather than linkage. These additional analyses strengthened the evidence for the causal relationships identified through our primary MR analysis.

### 2.8 Phenome-wide association analysis

To systematically evaluate the pleiotropic effects and potential adverse outcomes associated with our identified drug target, we conducted a comprehensive phenome-wide association study (PheWAS) through the AstraZeneca PheWAS Portal (https://azphewas.com/) (27). The PheWAS investigation offers valuable insights into both the complex genetic architecture underlying these traits and the therapeutic potential of the drug target, while simultaneously enabling systematic evaluation of its safety profile.

### 2.9 Drug prediction and evaluation of existing drugs

To evaluate the druggability potential of the identified genes, we employed the Enrichr tool (https://maayanlab.cloud/Enrichr/) to predict drugs or compounds for our identified gene using DSigDB database. Additionally, we interrogated established pharmacological databases including DrugBank (https://go.drugbank.com/) and the ChEMBL database (www.ebi.ac.uk/chembl/) to identify existing drugs targeting these genes and assess their repurposing potential.

### 2.10 Enzyme-linked immunosorbent assay for plasma RNASET2

The concentration of human RNASET2 in plasma samples from both AIT patient and control groups was quantified using a commercially available ELISA kit (MEIMIAN product #MM-65296H1, Jiangsu, China) according to the manufacturer’s instructions. Briefly, plasma samples were initially diluted 5-fold to ensure readings fell within the linear range of the standard curve. A series of known concentrations of recombinant RNASET2 protein were prepared to generate the standard curve. Then, 100 µL of each diluted standard, diluted plasma sample, and blank control were added to the appropriate wells of the pre-coated antibody plate and incubated for 30 minutes at 37°C. After incubation, the liquid was discarded, and each well was washed six times with the provided wash buffer. Subsequently, 100 µL of the detection antibody was added to each well and incubated for 30 minutes at 37°C. Following another wash cycle, 100 µL of substrate solution was added to each well and incubated at 37°C for 15 minutes for color development. The enzymatic reaction was stopped by adding stop solution, which changed the color from blue to yellow. The optical density (OD) of each well was immediately measured at 450 nm using a Spark microplate reader (Tecan, Männedorf, Switzerland). The average OD values of the blank control wells were subtracted from all standard and sample readings. A four-parameter logistic curve was fitted using the standard concentrations and their corresponding OD values to interpolate the exact RNASET2 concentration in each plasma sample.

### 2.11 Culture and treatment of thyrocyte spheroids

Human thyroid Nthy-ori-31 cells were grown in RPMI-1640 medium containing 10% FBS and 5 mM L-glutamine. Cells were seeded in 96-well Corning spheroid microplates at a density of 2,000 cells per well for 24 hours to allow spontaneous formation of three-dimensional (3D) spheroids. To induce an inflammatory response in the spheroids, 0.2 μg per well of synthetic dsRNA, i.e. poly(I:C) (InvivoGen product #tlrl-pic, Carlsbad, CA) was transfected into cells using jetPRIME transfection reagents (Polyplus, Illkirch, France) according to the manufacturer’s instructions. The following day, half of the culture medium was replaced with fresh medium containing increasing concentrations (0, 10, 100, and 1000 ng/mL) of recombinant human RNASET2 (MedChemExpress, Monmouth Junction, NJ). Twenty-four hours after RNASET2 treatment, the spheroids were subjected to further functional assays as described below.

### 2.12 Knockdown of RNASET2 using small interfering RNAs in human thyrocytes

Nthy-ori-31 cells were cultured as monolayers in 6-well plates and transfected with siRNAs targeting human RNASET2 using the jetPRIME transfection reagent (Polyplus) according to the manufacturer’s protocol. Three candidate siRNAs were tested: siRNA#1 (sense: 5′-GACAAUUGGUCAGAUAGAA(dT)(dT)-3′; anti-sense: 5′-UUCUAUCUGACCAAUUGUC(dT)(dT)-3′), siRNA#2 (sense: 5′- CGGAUUACUGGACAAUACA(dT)(dT)-3′; anti-sense: 5′-UGUAUUGUCCAGUAAUCCG(dT)(dT)-3′), and siRNA#3 (sense: 5′-CGCUCAACUCCCAGAAGAA(dT)(dT)-3′); anti-sense: 5′-UUCUUCUGGGAGUUGAGCG(dT)(dT)-3′). A negative control siRNA (siRNA_NC) was also tested. Forty-eight hours post-transfection, the cells were stimulated with poly(I:C) (1 μg per well). To evaluate the knockdown efficiency, total RNA was harvested 24 hours after poly(I:C) stimulation, and RNASET2 mRNA levels were quantified by real-time quantitative PCR. Secreted RNASET2 protein levels in culture supernatants were measure by ELISA. The siRNA demonstrating the highest knockdown efficiency was selected for all subsequent experiments. For the subsequent 3D spheroid assays, cells were harvested 24 hours after siRNA transfection and seeded into 96-well Corning spheroid microplates.

### 2.13 RNA extraction and real-time PCR analysis

Total RNA was extracted from cells using TRIzol (Invitrogen, Carlsbad, CA), and cDNA was synthesized using HiScript III RT SuperMix kit (Vazyme, Nanjing, China) according to the manufacturer’s instructions. Real-time PCR was performed using the QuantStudio 5 Real-Time PCR System (ThermoFisher Scientific, Waltham, MA) and the Fast SYBR Green Master Mix (ThermoFisher Scientific). The primers used in the study were: *GAPDH* forward, 5′-ACAGCAACAGGGTGGTGGAC-3′; *GAPDH* reverse, 5′-TTTGAGGGTGCAGCGAACTT-3′; *TNFA* forward, 5′-GACAAGCCTGTAGCCCATGT-3′; *TNFA* reverse, 5′-GACAAGCCTGTAGCCCATGT -3′; *IFNB* forward, 5′-TGCTCTCCTGTTGTGCTTCTCCAC-3′; *IFNB* reverse, 5′-CAATAGTCTCATTCCAGCCAGTGC-3′; *IL6* forward, 5′-CTCAATATTAGAGTCTCAACCCCCA-3′; *IL6* reverse, 5′-GAGAAGGCAACTGGACCGAA-3′; *IL1B* forward, 5′-ATGATGGCTTATTACAGTGGCAAT-3′; *IL1B* reverse, 5′-GTCGGAGATTCGTAGCTGGATG-3′; *RNASET2* forward, 5′-CTGGACCTCAACAGTGTGCT-3′; *RNASET2* reverse, 5′-CTTGGTGGAAGGCACTGGAT-3′. A total of 10 ng cDNA mixed with 10 μL 2× Fast SYBR Green Master Mix (ThermoFisher Scientific) was amplified by incubating for 20 minutes at 95°C, followed by 40 cycles of 3 seconds at 95°C and 30 seconds at 60°C. Real-time PCR analysis was carried out in triplicate and the relative mRNA expression levels were normalized against *GAPDH* levels.

### 2.14 Luminescent 3D cell viability assay

The cell viability of 3D spheroids was assessed using the CellTiter-Lumi™ Luminescent Cell Viability Assay Kit (Beyotime Biotechnology product #C0068S, Shanghai, China) according to the manufacturer’s instructions for 3D cultures. Briefly, following the desired treatment period, an equal volume of the pre-mixed CellTiter-Lumi™ detection reagent was directly added to each well of the 96-well spheroid microplate. The plate was then incubated for 5 minutes with shaking at room temperature to facilitate efficient cell lysis and subsequent reaction equilibrium. The cell lysis was then transfer to a white microplate for measurement of luminescence. Following a subsequent 10-minute incubation period at room temperature without shaking to stabilize the luminescent signal, the plate was read using a Spark multimode microplate reader (Tecan). The relative luminescence units (RLU) were recorded to determine the viability of spheroids.

### 2.15 Calcein-AM/propidium iodide (PI) live/dead viability/cytotoxicity assay

The viability/cytotoxicity of 3D spheroids was also assessed using the Beyo3D™ Calcein-AM/PI Cell Viability and Cytotoxicity Assay Kit (Beyotime Biotechnology product #C2035S) according to the manufacturer’s instructions. This assay simultaneously distinguishes live (labeled with calcein-AM) and dead (labeled with PI) cells based on intracellular esterase activity and plasma membrane integrity. Briefly, after treatment, the culture medium was gently aspirated, and the spheroids were washed once. The spheroids were then incubated with the working solution, prepared by diluting both Calcein-AM and PI in the provided assay buffer, for 45 minutes at 37 °C protected from light. Following incubation, the staining solution was carefully replaced with fresh assay buffer to reduce background fluorescence. The stained spheroids were immediately imaged using an Olympus APX100 imaging system (Olympus Corporation, Japan); the relative fluorescence units (RFU) were recorded using a Spark multimode microplate reader (Tecan) to determine the viability/cytotoxicity of spheroids.

### 2.16 Statistics

Statistical analyses of functional assays were conducted using GraphPad Prism 10 software (GraphPad Software Inc., San Diego, CA). The experiments were repeated at least three times. Significant differences were determined by unpaired Student’s t-test or ordinary one-way analysis of variance (ANOVA) followed by Dunnett’s *post-hoc* test. A P-value < 0.05 was considered statistically significant.

## 3 Results

### 3.1 MR analysis in discovery phase

**Figure 2:**
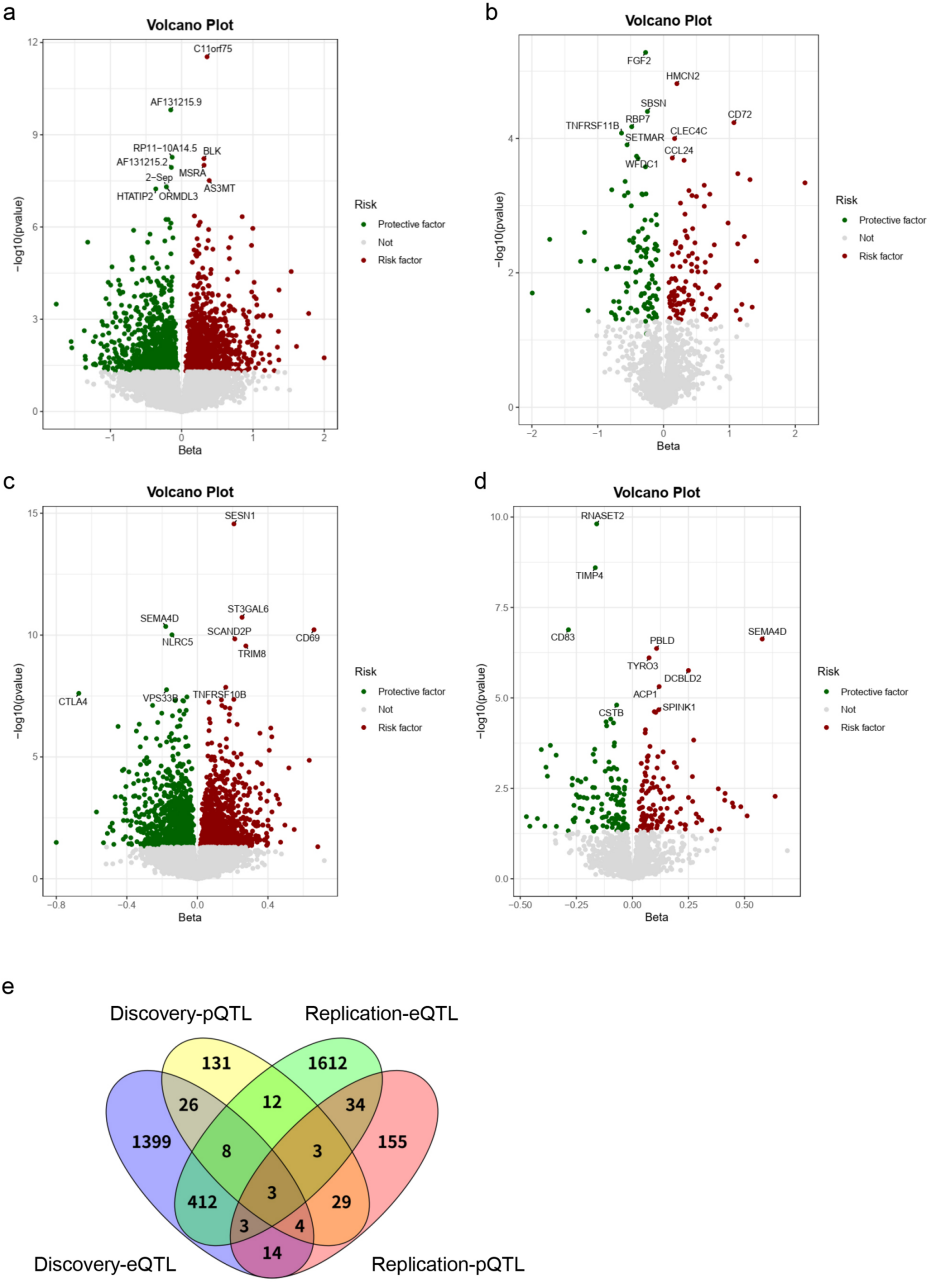
Three molecules were consistently identified to be causally associated with AIT risk. **(a, b)** Volcano plots for identified risk and protective eQTLs and pQTLs, respectively, for AIT in discovery phase. **(c, d)** Volcano plots for identified risk and protective eQTLs and pQTLs, respectively, for AIT in replication phase. **(e)** Venn plot shows that there were three molecules (CD40, RNASET2, FES) consistently validated across both discovery and replication phases.

Based on the selection criteria for IVs, we identified 1–145 independent IVs to investigate 15,695 cis-eQTLs and 1–152 IVs for 1,811 pQTLs. In the discovery phase using the FinnGen cohort’s AIT dataset, two-sample MR analysis (IVW method, P < 0.05) revealed 1,869 significant eQTLs including 956 protective factors (OR < 1) and 913 risk factor (OR > 1) (**Figure 2a**), and 216 pQTLs including 109 protective factors and 107 risk factors (**Figure 2b**). By intersecting the AIT-associated eQTL and pQTL, we discovered 31 overlapping molecules with consistent directions of impact on AIT, including 17 protective factors (FGF2, C4BPB, CD226, SSC5D, CHMP6, RBP7, DBI, CLEC1A, TNFSF14, GZMB, CRTAM, NIT2, ARG1, CD40, GSN, S100A12, RNASET2) and 14 risk factors (CLEC4C, CST3, FES, IMPA1, VSIG10, SPAG1, CDH2, MAPK9, EFCAB2, LGALS1, CANT1, IL17RB, CHIT1, PFKFB2).

### 3.2 MR analysis in replication phase

In the replication phase, we performed two-sample MR analysis using data from the IEU Open GWAS project to further examine the causal relationships between eQTLs/pQTLs and AIT. At a significance threshold of P < 0.05 (IVW method), we identified 2,087 significant eQTLs (1,019 protective factors and 1,068 risk factors) (**Figure 2c**) and 245 pQTLs (129 protective factors and 116 risk factors) (**Figure 2d**). Intersection analysis revealed 33 overlapping molecules with consistent effects on AIT, including 19 protective factors (GGT5, CD274, PDCD1LG2, SCARA5, CD83, PARP1, PKN3, CPVL, SWAP70, FCRLB, SHPK, RNASET2, CD40, ITIH4, PON2, CAPG, CSTB, SELL, SERPINE2) and 14 risk factors (DKK3, VNN2, CXCL5, FES, SH3GLB2, IL12RB1, FXYD5, DCBLD2, CLEC11A, F2R, COQ7, M6PR, TNFRSF10B, ACP1).

**Figure 3:**
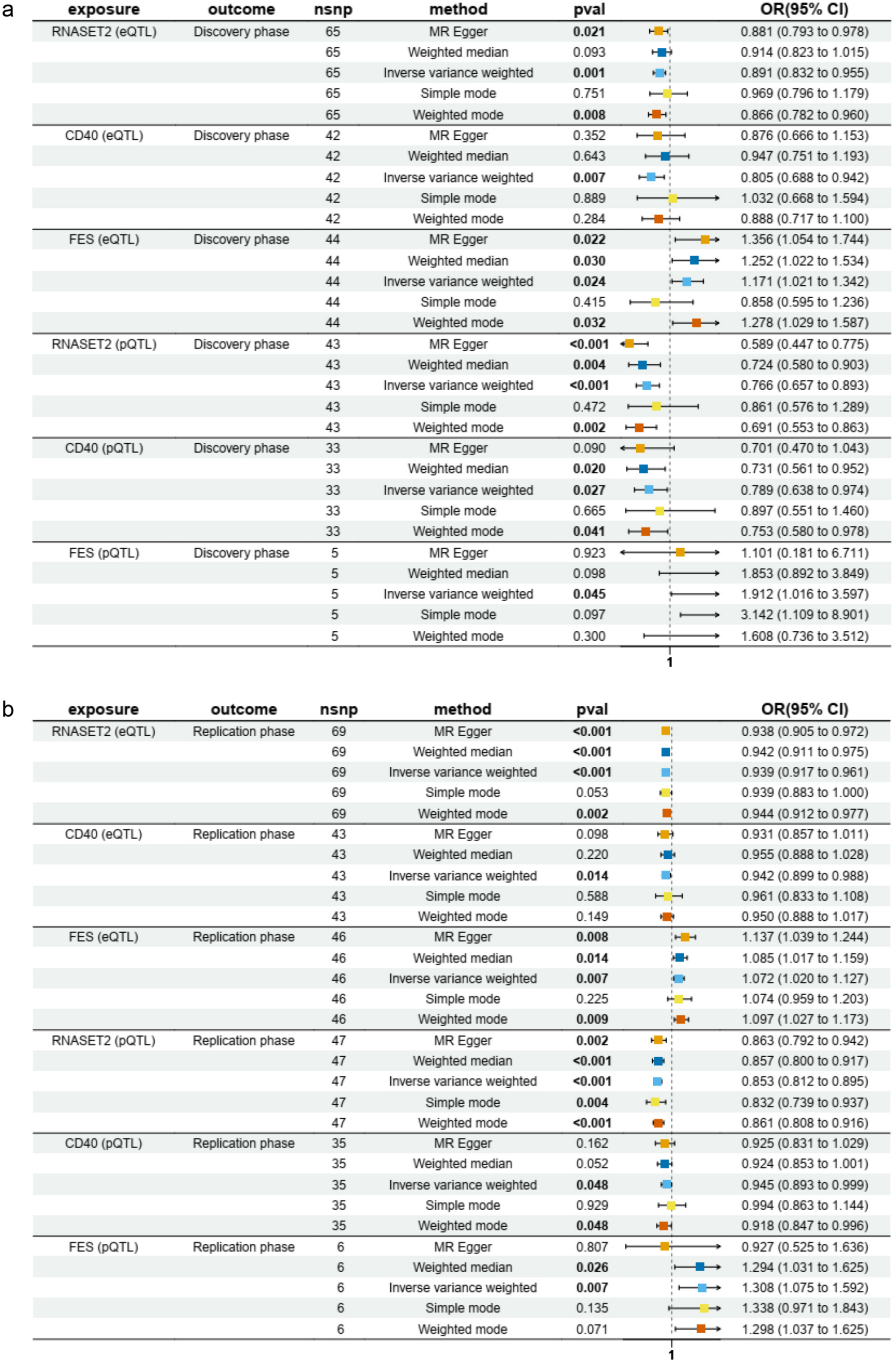
The forest plots to visualize protective effects of eQTL and pQTL of RNASET2 on AIT risk validated across both discovery **(a)** and replication **(b)** phases.

Notably, three molecules were consistently validated in both discovery and replication phases: CD40 and RNASET2 were associated with a reduced risk of AIT while FES was associated with an increased risk of AIT (**Figure 2e**). In particular, our multi-method validation approach, incorporating two to four additional analytical strategies beyond IVW, consistently identified RNASET2 as a protective factor against AIT across both discovery and replication phases (**Figure 3**, **Supplementary Table S1**). While sensitivity analyses generally supported the robustness of these findings, we observed certain methodological nuances: 1) The MR-Egger intercept test (though not MR-PRESSO) suggested potential horizontal pleiotropy for RNASET2 pQTL in the discovery phase, and 2) heterogeneity was detected among IV estimates of FES eQTL during replication (**Supplementary Table S1**).

### 3.3 SMR analysis and HEIDI test confirmed RNASET2 as a protective factor against AIT

**Figure 4:**
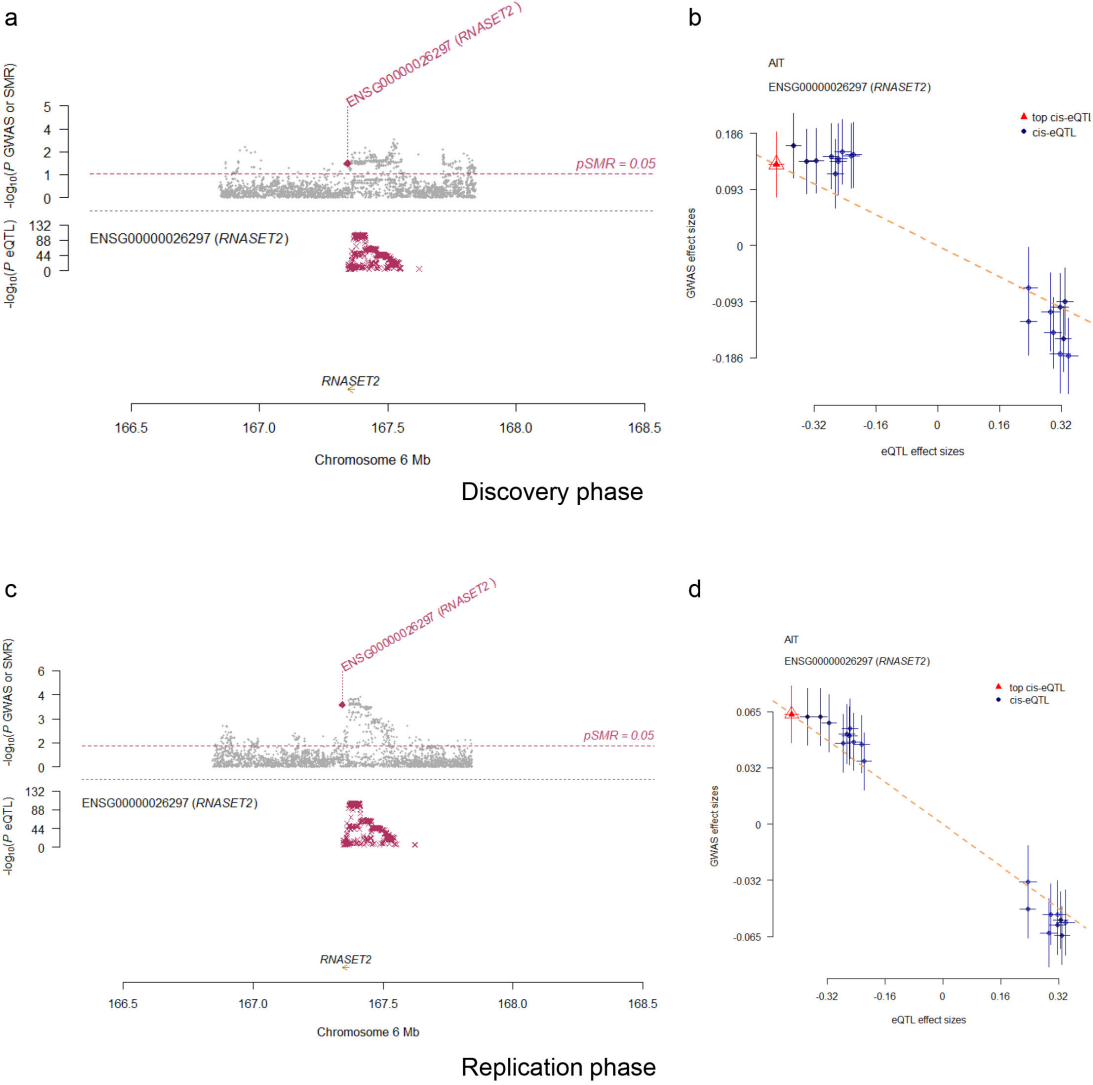
The SMR locus plots and effects plots for correlation of RNASET2 gene expression with AIT risk validated across both discovery **(a, b)** and replication **(c, d)** phases.

To further evaluate potential confounding due to pleiotropy and linkage disequilibrium, we performed SMR and HEIDI tests for RNASET2, CD40, and FES at the gene expression level. Among these candidates, only RNASET2 (**Table 1**) consistently passed both the SMR test (P < 0.05) and HEIDI test (P > 0.05), demonstrating robust evidence for a direct association with AIT. Locus and effect size plots from the SMR analysis are presented in **Figure 4**. These results corroborate our primary MR results and confirm the protective role of RNASET2 against AIT.

**Table 1:** SMR analysis and HEIDI test of causal relationships between AIT and gene expressions.

Phase	Gene	OR	OR (95% CI)	pSMR	pHEIDI
Discovery	RNASET2	0.7280	0.5655-0.9371	0.0137	0.1207
	FES	1.2251	0.4214-3.5616	0.7093	0.9882
	CD40	0.8622	0.6437-1.1549	0.3201	0.5288
Replication	RNASET2	0.8608	0.7972- 0.9296	0.0001	0.5880
	FES	1.1074	0.7873-1.5578	0.5576	0.2197
	CD40	0.9699	0.8868-1.0610	0.5054	0.2946

### 3.4 mQTL MR analysis suggest DNA methylation of RNASET2 as a risk factor of AIT

**Figure 5:**
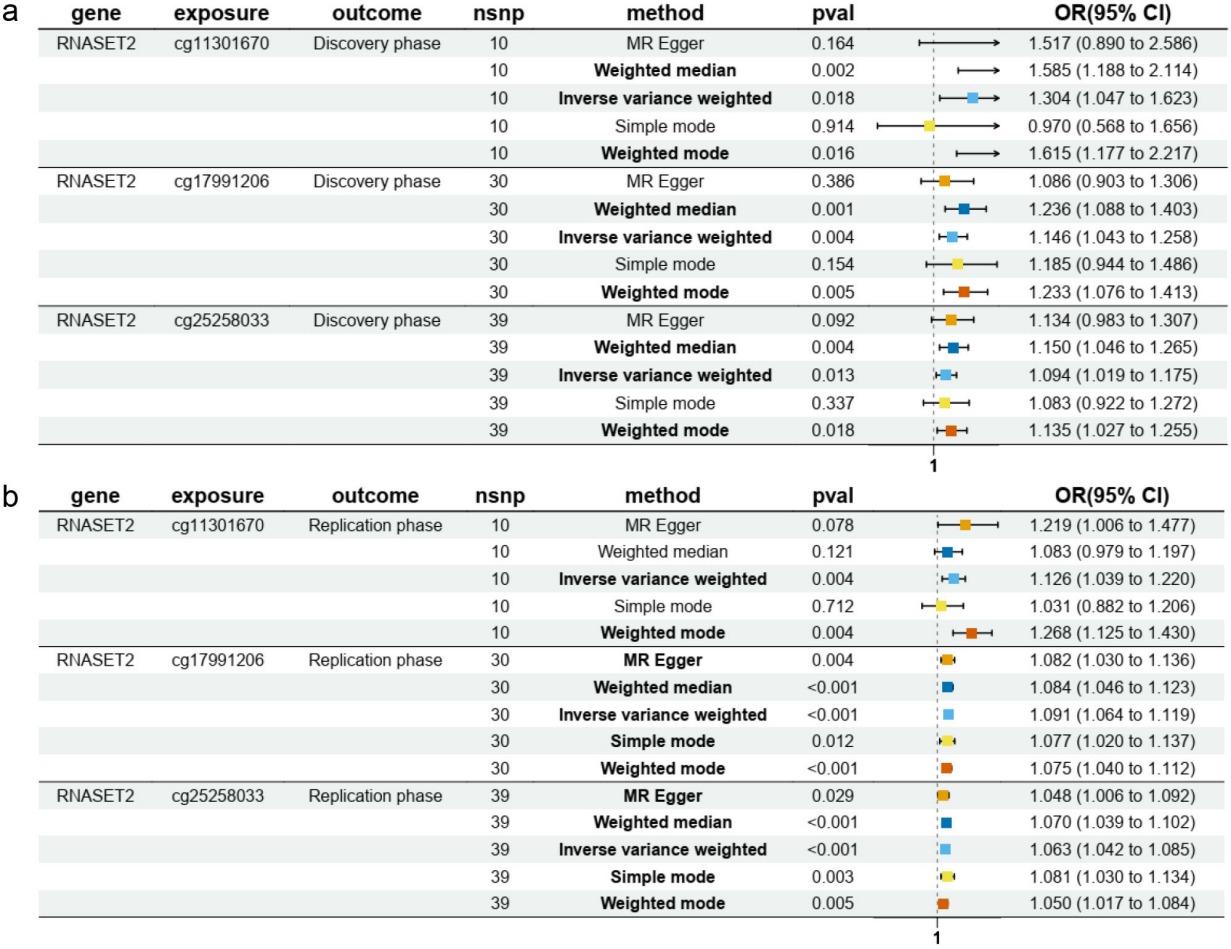
The forest plots to visualize the effects of three mQTLs of RNASET2 (cg11301670, cg17991206, and cg25258033) on AIT risk validated across both discovery **(a)** and replication **(b)** phases.

DNA methylation is an epigenetic process involving the addition of methyl groups to DNA, which can modulate (typically suppress) gene activity without altering the sequence (28). Investigating genetic influences on DNA methylation (DNAm) offers insights into gene regulation and disease mechanisms (14). To assess whether *RNASET2* methylation influences AIT risk, we conducted mQTL-specific MR analysis. We retrieved summary data for 11 *RNASET2*-associated mQTLs from the GoDMC analysis, a large-scale study that systematically mapped *cis*- and *trans*-acting genetic influences on DNA methylation using whole-blood samples from 27,750 European individuals across 36 cohorts (14). Based on the predefined IV selection criteria, we identified 1–39 independent IVs to analyze 7 mQTLs with two independent AIT GWAS datasets (discovery and validation cohorts). At the significance threshold of 0.05 (IVW method), 3 mQTLs (cg11301670, cg17991206, and cg25258033) were consistently associated with an increased risk of AIT across both discovery and replication phases (**Figure 5**). These associations were further supported by at least one additional validation approach (**Figure 5**, **Supplementary Table S2**). No horizontal pleiotropy or heterogeneity was indicated by the sensitivity analyses (**Supplementary Table S2**). To ensure the robustness of our results, scatter plots, funnel plots, and forest plots for MR leave-one-out sensitivity analysis were further generated (**Supplementary Figures S1**, **S2**), confirming that the estimated effects of eQTL, pQTL, and mQTL of RNASET2 on AIT were unlikely to be driven by any single genetic instrument. Characteristics of significant SNPs with genome-wide associations for eQTL, pQTL, and mQTL of RNASET2 with AIT risk were summarized in **Supplementary Tables S3-S4**.

### 3.5 PheWAS and candidate drug prediction

Through the PheWAS Portal, we performed phenome-wide MR to assess potential side effects associated with targeting RNASET2. The analysis revealed no significant associations between RNASET2 and other phenotypes in the PheWAS Portal (**Supplementary Figure S3**) at the genome-wide significance threshold (P < 5 × 10^-8^). These findings support the robustness of our results and suggest that therapeutic targeting of RNASET2 is unlikely to lead to adverse drug reactions or unintended horizontal pleiotropic effects. To identify potential drug candidates targeting RNASET2 for the treatment of AIT, we first screened established pharmacological databases. However, no currently approved or investigational drugs targeting RNASET2 were documented in either DrugBank or ChEMBL. We therefore searched for possible drugs targeting RNASET2 using the DSigDB drug database on Enrichr. The top 10 predicted drugs associated with RNASET2 were summarized in **Table 2**.

**Table 2:** Drug prediction for RNASET2 using DSigDB database.

Index	Name	P-value	Adjusted P-value	Odds ratio	Combined score
1	ionomycin (CTD 00007090)	0.0056	0.05035	19888	103119.31
2	daunorubicin (PC3 UP)	0.00705	0.05035	19859	98396.17
3	Pyrrolidine dithiocarbamate (CTD 00001021)	0.00795	0.05035	19841	95923.19
4	theophylline (CTD 00006862)	0.0236	0.09177	19528	73161.95
5	Phorbol 12-myristate 13-acetate (CTD 00006852)	0.02415	0.09177	19517	72671.11
6	Vitinoin (CTD 00007069)	0.039	0.1024	19220	62353.5
7	Caspan (CTD 00000180)	0.0404	0.1024	19192	61585.79
8	lycorine (HL60 DOWN)	0.0431	0.1024	19138	60174.41
9	genistein (CTD 00007324)	0.06155	0.1219	18769	52326.27
10	Arsenenous acid (CTD 00000922)	0.06415	0.1219	18717	51406.89

### 3.6 Validation of therapeutic effects of recombinant RNASET2 in human thyrocyte spheroids

**Figure 6:**
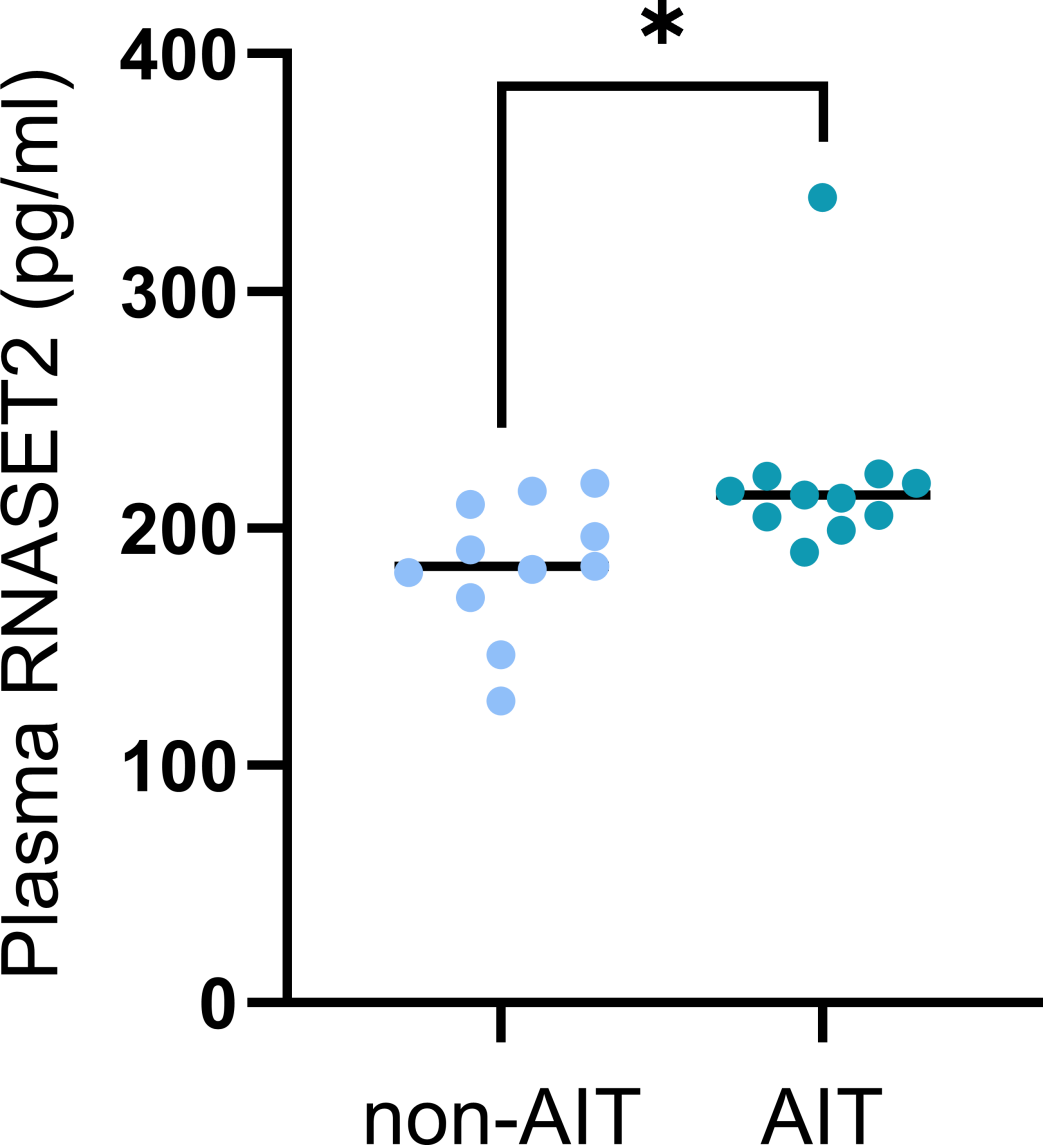
Plasma RNASET2 levels were elevated in AIT patients. Plasma RNASET2 levels in AIT patients (n = 12) and non-AIT controls (n = 12) were quantified through ELISA. Data are presented as individual values with the median. A significant difference between groups was determined using an unpaired two-tailed Student’s t-test (*P < 0.05).

In the experiment validation phase, we first assessed the plasma RNASET2 levels in patients with AIT (n = 12) and non-AIT controls (n = 12) to determine whether a significant difference existed between the two groups. Although the MR study consistently identified RNASET2 as a protective factor against AIT, we observed slightly elevated plasma RNASET2 levels were in AIT patients (**Figure 6**).

**Figure 7:**
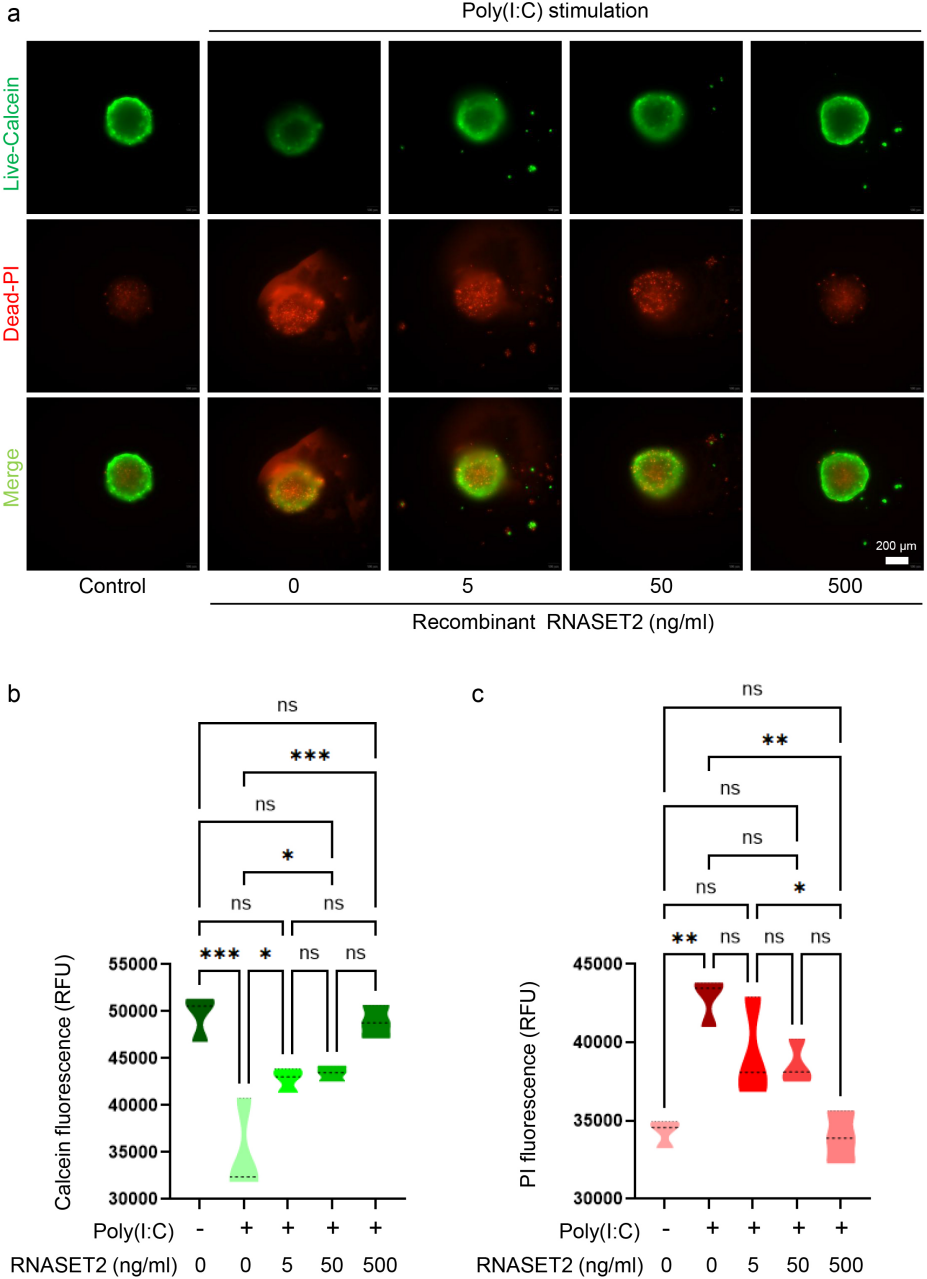
Recombinant RNASET2 attenuates poly(I:C)-induced cell death in thyrocyte spheroids. Thyrocyte spheroids were transfected with poly(I:C) to simulate inflammatory injury and then treated with increasing concentrations of recombinant RNASET2. Poly(I:C) transfection induced significant cell death, as indicated by increased PI staining and reduced calcein AM signal. Recombinant RNASET2 treatment inhibited poly(I:C)-induced cell death in a dose-dependent manner, as shown by representative fluorescence images **(a)** and quantitative analysis of fluorescence intensity **(b, c)**. Data are presented as violin plots. Statistical significance was determined by ordinary one-way ANOVA followed by Dunnett’s *post-hoc* test (*P < 0.05, **P < 0.01, ***P < 0.001, ns: not significant).

**Figure 8:**
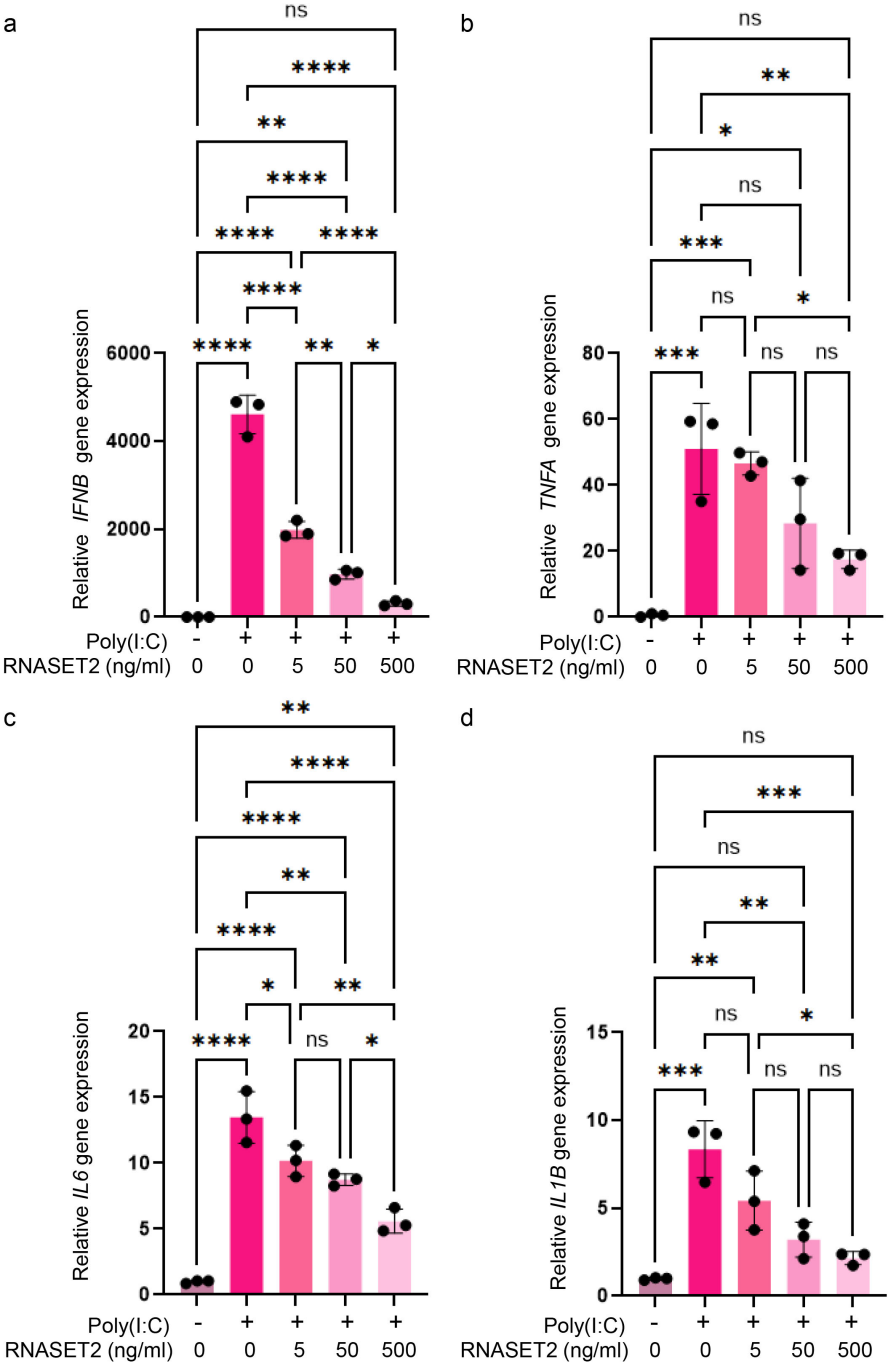
Recombinant RNASET2 suppresses poly(I:C)-induced gene expression of inflammatory cytokines in thyrocyte spheroids. Thyrocyte spheroids were transfected with poly(I:C) and subsequently treated with increasing concentrations of recombinant RNASET2. **(a–d)** Poly(I:C) transfection significantly upregulated the mRNA expression of inflammatory cytokines (*IFNB*: IFN-β, *TNFA*: TNF-α, *IL6*: IL-6, *IL1B*: IL-1β) which was suppressed by RNASET2 treatment in a dose-dependent manner. The relative mRNA expression levels were normalized against *GAPDH* levels. Data are presented as mean ± SEM. A significant difference was determined by ordinary one-way analysis of variance (ANOVA) followed by Dunnet’s *post-hoc* test and multiple comparison tests (*P < 0.05, **P < 0.01, ***P < 0.001, ****P < 0.0001, ns: not significant).

Despite this observation, we further investigated whether recombinant RNASET2 could affect AIT-related pathology using a novel model: the inflammatory human thyrocyte spheroids induced by transfection with poly(I:C) fragments. Human thyroid Nthy-ori-31 cells were cultured in ultra-low attachment plates to facilitate the natural formation of 3D spheroids. To mimic an inflammatory phenotype representative of AIT, the spheroids were stimulated via transfection with poly(I:C) fragments, which we have previously shown to induce robust inflammatory responses and cell death in monolayer culture of thyrocytes (29). Following transfection, spheroids were treated with increasing concentrations of recombinant RNASET2 for 24 hours and subsequently evaluated. Luminescent 3D cell viability assays revealed that recombinant RNASET2 treatment (5–500 ng/mL) by itself did not significantly affect thyrocyte spheroid viability (**Supplementary Figure S4a**). Consistent with this, a live/dead 3D viability/cytotoxicity assay using calcein AM and propidium iodide (PI) staining confirmed the absence of cytotoxic effects within the tested dose range. Neither fluorescence microscopy imaging (**Supplementary Figure S4b**) nor quantitative fluorescence measurements using a microplate reader showed significant differences between treated and untreated spheroids (**Supplementary Figures S4c, d**). Similar to the previously established inflammatory models (29), poly(I:C) transfection induced substantial cell death in thyrocyte spheroids, as evidenced by increased PI-positive staining and reduced calcein AM-positive signals (**Figure 7**). Recombinant RNASET2 treatment inhibited this poly(I:C)-induced cell death in a dose-dependent manner, as demonstrated by both qualitative microscopic examination (**Figure 7a**) and quantitative fluorescence intensity measurements (**Figures 7b, c**). In parallel, unstimulated thyrocytes spheroids exhibited minimal gene expressions of inflammatory cytokines such as interferon-β (IFN-β), tumor necrosis factor α (TNF-α), interleukin-6 (IL-6), and IL-1β. Poly(I:C) stimulation markedly upregulated gene expressions of these cytokines, an effect that was significantly suppressed by recombinant RNASET2 in a dose-dependent fashion (**Figure 8**).

**Figure 9:**
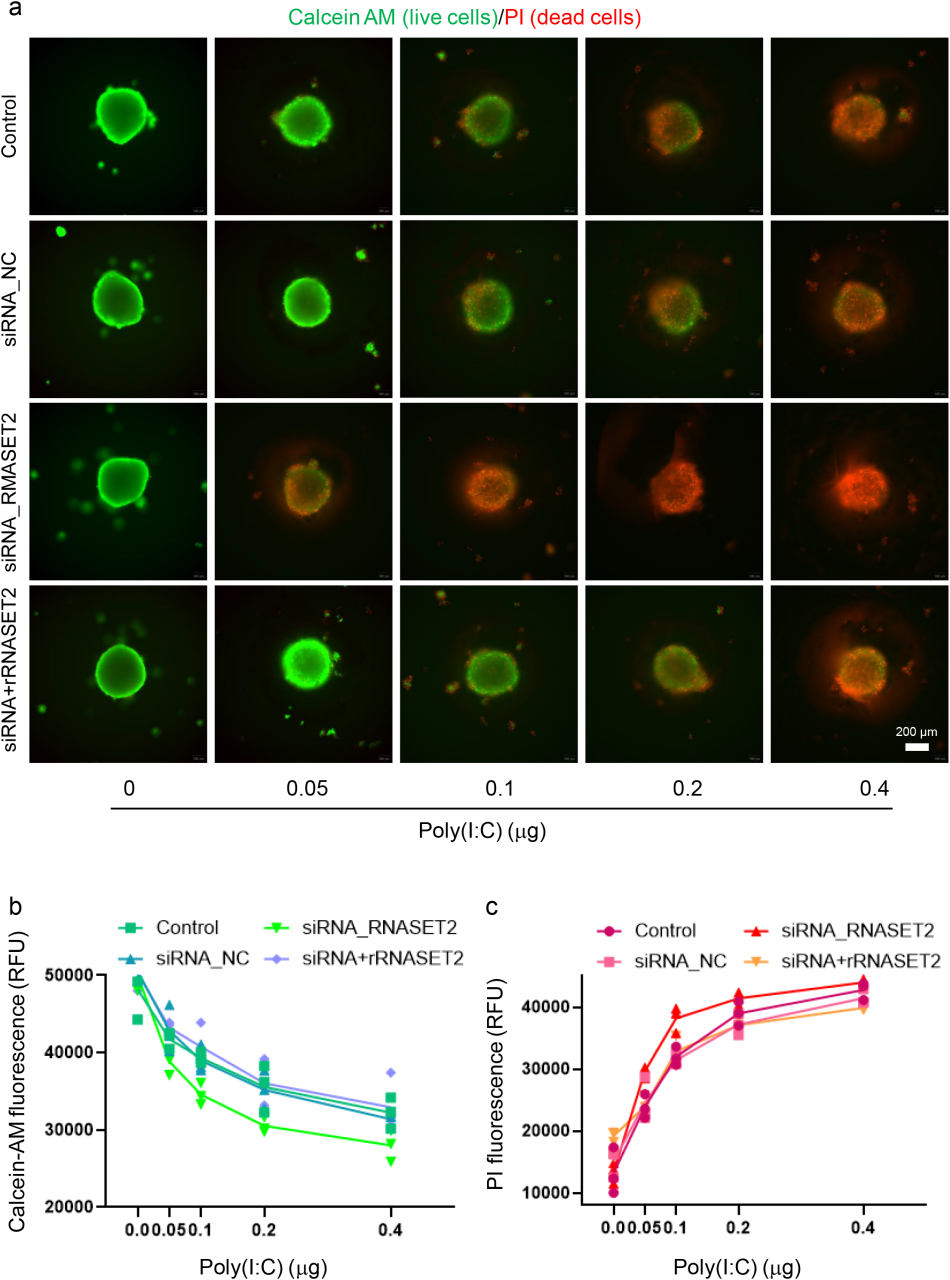
Enhanced susceptibility to poly(I:C)-induced cell death in RNASET2-knockdown thyrocyte spheroids was rescued by recombinant RNASET2. Thyrocyte spheroids, with or without RNASET2 knockdown, were stimulated with increasing doses of poly(I:C) (0.05-0.4 µg per spheroid) for 24 hours in the absence or presence of 500 ng/ml recombinant RNASET2 treatment. **(a)** Merged fluorescence micrographs showing viability (Calcein-AM, green) and cell death (PI, red) in spheroids. RNASET2-knockdown spheroids exhibited heightened cell death at all doses tested, which was reversed by recombinant RNASET2 supplementation. Individual fluorescence channels were presented in **Supplementary Figure S6**. **(b, c)** Quantification of fluorescence intensity of calein-AM and PI. Triplicate data points are shown. Control: non-transfected spheroids; siRNA_NC: spheroids transfected with the negative control siRNA; siRNA_RNASET2: RNASET2-knockdown spheroids; siRNA+rRNASET: RNASET2-knockdown spheroids supplemented with recombinant RNASET2 protein.

**Figure 10:**
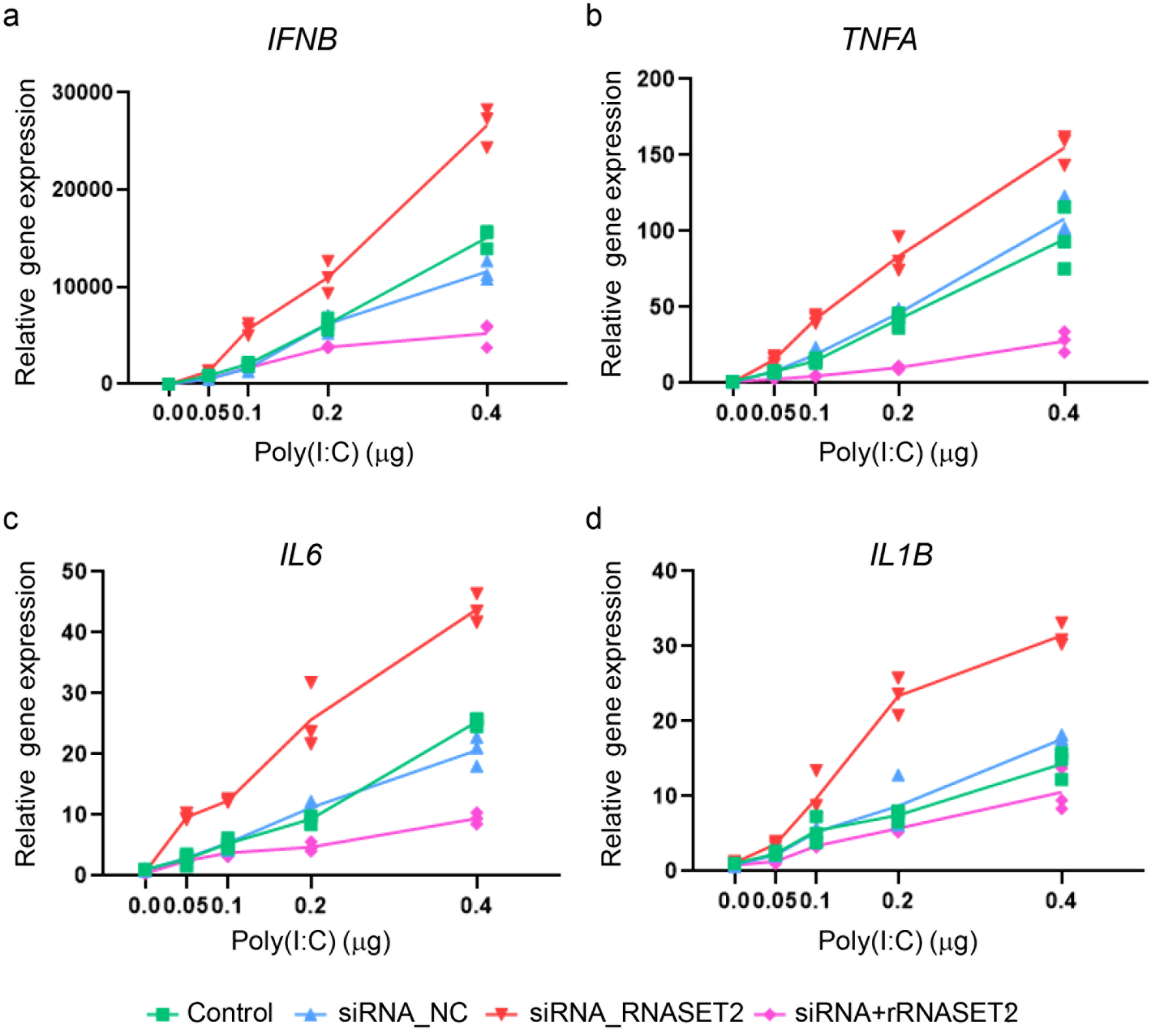
Recombinant RNASET2 reversed elevated inflammatory cytokine expression in RNASET2-knockdown thyrocyte spheroids after poly(I:C) stimulation. Thyrocyte spheroids, with or without, RNASET2 knockdown were stimulated with increasing doses of poly(I:C) (0.05-0.4 µg per spheroid) for 24 hours, in the absence or presence of 500 ng/ml recombinant RNASET2. **(a–d)** RNASET2-knockdown spheroids exhibited higher mRNA levels of inflammatory cytokines (*IFNB*: IFN-β; *TNFA*: TNF-α; *IL6*: IL-6; *IL1B*: IL-1β) across all poly(I:C) doses tested, an effect rescued by recombinant RNASET2 supplementation. Relative mRNA expression was normalized to GAPDH. Triplicate data points are shown. Control: non-transfected spheroids; siRNA_NC: spheroids transfected with the negative control siRNA; siRNA_RNASET2: RNASET2-knockdown spheroids; siRNA+rRNASET: RNASET2-knockdown spheroids supplemented with recombinant RNASET2 protein.

Endogenous RNASET2 expression was moderately upregulated in thyrocyte spheroids upon poly(I:C) stimulation, both at mRNA and secreted protein levels (**Supplementary Figure S5**), which aligns with the mildly elevated plasma RNASET2 observed in AIT patients (**Figure 6**). To explore RNASET2’s protective role in thyroid inflammation, we performed siRNA-mediated knockdown in human thyrocytes and evaluated function in the 3D spheroid model. Among three candidate siRNAs, siRNA#2 was selected for subsequent experiments due to its highest knockdown efficiency, achieving an approximately 90% and 80% reduction, respectively, in RNASET2 mRNA expression and protein secretion of poly(I:C)-stimulated thyrocytes (**Supplementary Figure S6**). Compared to non-transfected spheroids or those transfected with the negative control siRNA (siRNA_NC), RNASET2-knockdown spheroids exhibited increased susceptibility to poly(I:C)-induced cell death across all tested doses (0.05-0.4 μg per spheroid), as demonstrated by 3D calcein-AM/PI staining (**Figure 9**, **Supplementary Figure S7**). Meanwhile, these knockdown spheroids exhibited elevated inflammatory cytokine mRNA expression at all poly(I:C) concentrations compared to controls (**Figure 10**). Importantly, supplementation with recombinant RNASET2 protein rescued both the cell death and pro-inflammatory phenotypes in knockdown spheroids (**Figures 9**, **10**). Together, these findings demonstrate that RNASET2 exerts a protective effect against inflammation and cell death in thyrocytes. Recombinant RNASET2 effectively mitigates poly(I:C)-induced inflammatory responses and cell death in human thyrocyte spheroids, supporting its potential as a therapeutic candidate for AIT.

## 4 Discussion

AIT ranks among the most prevalent autoimmune disorders. While lifelong hormone replacement therapy effectively manages resultant hypothyroidism, the underlying autoimmune response and thyroid destruction remain untreated. This critical therapeutic gap underscores the urgent need for novel AIT treatment. By integrating GWAS data analyzing cis-eQTLs, pQTLs, and mQTLs with two independent AIT GWAS datasets, we identified RNASET2 as a novel causal therapeutic target for AIT. Moreover, we measured plasma RNASET2 concentrations in both AIT patients and non-AIT control subjects and further assessed the pharmacological actions of RNASET2 using a novel human inflammatory thyrocyte spheroid model. This approach enabled functional validation of its therapeutic potential within a physiologically relevant environment.

AIT develops through a complex interplay of genetic susceptibility and environmental triggers that disrupt immune tolerance to thyroid antigens. The pathogenesis involves antigen-presenting cells activating autoreactive T cells, leading to helper T cells (Th1/Th17)-dominated immune responses that drive inflammation (30), while defective regulatory T (Treg) cell function fails to suppress the autoimmune attack (31). Concurrently, pro-inflammatory M1 macrophages infiltrate the thyroid, releasing cytokines that perpetuate tissue damage, while insufficient M2 macrophage activity impairs tissue repair (32). This immune dysregulation, combined with genetic variants affecting immune pathways (33), creates a self-perpetuating cycle of chronic inflammation and progressive thyroid dysfunction. In addition to lymphocyte dysfunction, excessive activation of the innate immune response in thyrocytes may also contribute to the pathogenesis of AIT (34).

The human *RNASET2* gene encodes ribonuclease T2 (RNAse T2), the sole characterized member of the Rh/T2/S family of acid hydrolases in humans (35). This secreted ribonuclease, capable of RNA degradation, has been implicated in diverse biological processes, including cancer, inflammation, and autoimmune disorders (36). Intriguingly, RNASET2 exhibits context-dependent roles, displaying both pro- and anti-inflammatory effects in observational studies. Within the tumor stroma, RNASET2 functions as an endogenous “alarmin,” triggering innate immune responses by recruiting M1-polarized macrophages (37). Supporting this, overexpression of murine *Rnaset2* in a syngeneic colon carcinoma model shifted the intratumoral macrophage balance toward M1 polarization, enhancing anti-tumor CD8+ T cell infiltration and suppressing tumor growth (38). Beyond cancer, RNASET2 is essential for macrophage activation via toll-like receptor 8 (TLR8), promoting Th1 immune responses against intracellular pathogens (39). TLRs are endolysosomal RNA sensors activated by processed RNA. RNase T2 cleaves long RNAs into TLR-stimulating fragments but also prevents aberrant TLR activation, as patients deficient in RNase T2 suffer from neuroinflammation. *Rnaset2* deficiency triggers TLR13-dependent neuroinflammation and splenomegaly, even in germ-free mice, establishing RNase T2 as a checkpoint of TLR13-mediated autoinflammation (40). Thus, RNASET2 emerges as a pleiotropic immune modulator, orchestrating defense mechanisms against malignant transformation, microbial invasion, and autoinflammation through its dual roles in innate and adaptive immunity.

GWAS has also identified RNASET2 as a susceptibility locus for multiple inflammatory and autoimmune conditions, such as vitiligo (41), rheumatoid arthritis (42), Graves’ disease (43), and Crohn’s disease (44). Intriguingly, our study reveals RNASET2 as a novel protective factor in AIT, echoing its previously reported protective function in Crohn’s disease (CD). In CD pathogenesis, RNASET2 is unique among disease-risk genes, exhibiting > 5-fold downregulation in interferon-γ (IFN-γ)-secreting T cell subsets (45). Mechanistically, TL1A (a cytokine of the tumor necrosis factor superfamily) -mediated T cell stimulation leads to RNASET2 suppression, while disease-associated RNASET2 variants correlate with both reduced RNASET2 expression and enhanced IFN-γ production in T cells of CD patients (45). Clinically, RNASET2 disease-risk SNPs are associated with therapeutic failure of anti-tumor necrosis factor (TNF) therapy and overall severity of CD (46). CD patients requiring surgical intervention show significantly reduced circulating RNASET2 protein levels compared to healthy controls (46). Notably, surgical resection of inflamed tissue restores physiological RNASET2 concentrations, suggesting a feedback between inflammation and RNASET2 depletion (46). Functional studies reveal that RNASET2 overexpression or recombinant protein treatment can attenuate pro-inflammatory IFN-γ secretion in activated T cells (46). Similar to its emerging role in CD therapy, RNASET2 augmentation may represent a novel therapeutic avenue for AIT, as suggested by our findings. Notably, RNASET2 exhibits cell- and tissue-specific regulatory patterns that may explain its context-dependent immunomodulatory effects. In activated T cells, reduced RNASET2 expression correlates with increased IFN-γ production, while in granulocytes, elevated RNASET2 levels promote innate immune activation and macrophage recruitment (47). This dichotomous regulation likely underlies RNASET2’s capacity to mediate both pro- and anti-inflammatory responses depending on the cellular microenvironment.

Intriguingly, three mQTLs of *RNASET2* were consistently identified as risk factors for AIT in our study. This observation contrasts with the finding that RNASET2 eQTLs and pQTLs are protective against AIT, but the discrepancy may reflect the suppressive role of DNA methylation on gene expression (28). DNA methylation typically downregulates transcription either by recruiting repressive protein complexes or by blocking transcription factor binding (28). Supporting this mechanism, RNASET2 hypermethylation has been linked to its reduced expression in peripheral and mucosal tissues of CD patients, correlating with disease severity (45). Further evidence highlights the regulatory complexity of RNASET2: its CD-risk variant resides in chromatin regions enriched for T-cell-specific enhancer and promoter marks, and its transcription depends on cooperative interactions between *cis*- and *trans*-regulatory elements (46). These interactions govern transcription factor binding and appear sensitive to DNA methylation (46). Thus, RNASET2 expression is regulated by a complex, multilevel interplay of transcriptional and post-transcriptional mechanisms, which ultimately drives divergent cellular phenotype (46).

Human recombinant RNASET2 has demonstrated efficacy in suppressing tumor growth, infection, and inflammatory responses in several disease models, highlighting its potential as a soluble therapeutic agent (36). Our PheWAS analysis further supports its druggability, revealing minimal side effects and a low likelihood of pleiotropy-related bias, a critical advantage given the challenges of adverse drug effects in clinical trials. To contextualize RNASET2’s therapeutic potential, we referenced Finan et al. (15), which classifies druggable genes into three categories: 1) 1,427 genes targeted by approved drugs or clinical candidates; 2) 682 genes with bioactive small-molecule binders and ≥ 50% similarity to approved targets; 3) 2,370 genes encoding secreted/extracellular proteins or key members of druggable families. RNASET2 falls into the third category, which includes less characterized but biologically plausible targets (15). Using the DSigDB database (Enrichr), we identified potential RNASET2-targeting drugs and prioritized those that upregulate its expression, given its protective role in AIT. Genistein, a soy-derived isoflavone with reported anti-cancer, anti-inflammatory, and antioxidant properties, ranked among the top 10 predictions. Soy isoflavones induced a 4.5-fold upregulation of *RNASET2* in breast tissue of ovariectomized rats (48), suggesting a plausible mechanism for therapeutic effects. Whether genistein or soy supplementation could ameliorate AIT via RNASET2 upregulation warrants further investigation.

The findings from this study present a seemingly paradoxical scenario: while our large-data analysis indicates that genetically elevated RNASET2 confers a protective causal effect against AIT, we found that plasma levels of RNASET2 are significantly higher in AIT patients compared to non-AIT controls. However, this apparent contradiction is not uncommon in molecular medicine and can be reconciled through several biologically plausible mechanisms that reflect the complex interplay between genetic predisposition, dynamic disease processes, and compensatory physiological responses. A primary explanation for this phenomenon is the compensatory anti-inflammatory or cytoprotective response hypothesis. In this model, the elevated circulating RNASET2 is not a driver of pathology but rather a reactive upregulation by the body to counteract ongoing inflammation and tissue damage. AIT is characterized by lymphocytic infiltration, thyrocyte apoptosis, and production of pro-inflammatory cytokines. Our functional studies directly support this compensatory role. We found that endogenous RNASET2 expression in thyrocyte spheroids is moderately upregulated in response to poly(I:C) stimulation. More importantly, knockdown of RNASET2 rendered thyrocyte spheroids significantly more susceptible to inflammatory cell death and enhanced their expression of pro-inflammatory cytokines, while supplementation with recombinant RNASET2 protein rescued this vulnerable phenotype (**Figures 9**, **10**; **Supplementary Figure S7**). These results establish that RNASET2 possesses potent anti-apoptotic and anti-inflammatory properties within the thyroid cellular context. Thus, the MR data identifies the baseline genetic potential for protection, while the elevated plasma levels in patients reflect the system’s active, albeit insufficient, attempt to mitigate the inflammatory insult. Another compelling explanation is cellular leakage due to tissue destruction. RNASET2 may be constitutively expressed at high levels within thyroid follicular cells under normal conditions, where it performs housekeeping functions. During AIT, widespread immune-mediated damage and apoptosis of thyrocytes could lead to the release of intracellular contents, including RNASET2, into the circulation. Consequently, the increased plasma levels might not stem from active secretion but rather passively reflect the degree of tissue breakdown. This would position plasma RNASET2 not as a pathogenic agent but as a biomarker of disease activity and cellular injury. The timing and heterogeneity of patient sampling also offer critical insight. Our cross-sectional measurement captures RNASET2 levels at a single point in the disease course. It is possible that levels fluctuate dynamically—rising acutely during flares of autoimmunity and subsiding during remission. The MR estimate, on the other hand, reflects the lifelong influence of genetic predisposition. Therefore, we may be observing a snapshot of an acute-phase response rather than the steady-state level that governs long-term risk. Finally, unmeasured confounding factors could contribute to the observed elevation. For instance, systemic inflammatory mediators in AIT might directly stimulate the expression and release of RNASET2 from immune or endothelial cells outside the thyroid. This would create a non-causal association between AIT disease activity and RNASET2 plasma levels, independent of its role in thyroid protection. The MR approach, by leveraging genetic variants fixed at birth, helps circumvent such reverse causation and confounding, thus uncovering the true causal protective nature of RNASET2. Therefore, the elevation of plasma RNASET2 in AIT patients does not necessarily invalidate its protective role as indicated by MR; instead, it illuminates the complexity of its biology in a dynamic disease context. It likely represents a combination of a concerted compensatory effort by the organism to limit tissue damage and a consequence of active tissue destruction. Future longitudinal studies measuring RNASET2 levels before and after disease onset, combined with analysis of its tissue-specific expression and receptor activity, will be essential to distinguish between these mechanisms.

The observed dose-dependent anti-apoptotic and anti-inflammatory effects, as demonstrated in our thyrocyte spheroid model, position RNASET2 as a compelling candidate for therapeutic intervention in AIT. Current treatments for AIT, such as levothyroxine replacement, manage hypothyroidism but do not halt the underlying autoimmune process. By targeting the inflammatory cascade and thyrocyte apoptosis, RNASET2 offers a potential disease-modifying approach. Its ability to downregulate pro-inflammatory cytokines (e.g. IFN-β, TNF-α, IL-6, and IL-1β) suggests a broad immunomodulatory function, possibly through the inhibition of NF-κB or JAK-STAT signaling pathways, which are commonly activated in thyroid autoimmunity. Our rescue experiments with recombinant RNASET2 protein (**Figures 9**, **10**; **Supplementary Figure S7**) provide direct preclinical evidence for its therapeutic efficacy at the cellular level. Furthermore, the lack of cytotoxicity at effective concentrations supports its biological safety, a critical consideration for translational development. Future studies should explore its efficacy *in vivo*, particularly in preclinical models of AIT, and consider delivery mechanisms such as targeted thyroid-specific expression or sustained-release formulations to maximize local effects and minimize systemic exposure.

A key innovation of this study is the implementation of a 3D human thyrocyte spheroid model challenged with poly(I:C) to simulate thyroid inflammation. The 3D spheroid system better recapitulates critical features of the thyroid microenvironment, thereby providing a more authentic platform for studying immune-mediated damage. The use of poly(I:C)—a double-stranded RNA analog—to trigger innate immune activation effectively mimics viral-induced inflammation, a known trigger or exacerbating factor in AIT. This model allows for real-time assessment of cytokine dynamics, cell viability, and treatment responses in a tissue-like context, enhancing the translational validity of the findings. Its application extends beyond AIT to other thyroid disorders, such as viral thyroiditis or thyroid cancer-related inflammation, and might be adapted for high-throughput drug screening.

While our study provides solid evidence for RNASET2 as a therapeutic target in AIT, several limitations should be acknowledged. First, the large-scale data used in this study were derived from European populations, which may limit the generalizability of our findings to other ethnic groups. Second, our analyses primarily relied on blood-derived QTL data, which may not fully capture tissue-specific regulatory mechanisms in the thyroid gland. Third, the observational nature of GWAS data means that despite rigorous MR approaches, residual confounding cannot be completely ruled out. Fourth, the sample sizes of both the AIT patient and non-AIT control groups were relatively small, which may limit the statistical power and generalizability of the findings. Therefore, further validation in larger, independent cohorts is necessary to confirm these results. Moreover, *in vivo* studies are essential to substantiate the protective effects of RNASET2 observed in the thyrocyte spheroid model and to assess its physiological relevance in a whole-organism context. Also, given the potential pleiotropic functions of RNASET2 in other biological pathways, future preclinical studies should carefully evaluate its broader systemic effects to fully understand its efficacy and safety profile. These limitations highlight important considerations for translating our findings into clinical applications.

## 5 Conclusions

Through integrative genomic and epigenomic analyses, we have established RNASET2 as a causal protective factor in AIT. These findings were further corroborated by an *in vitro* inflammatory thyrocyte spheroid model, which recapitulated the potent anti-apoptotic and anti-inflammatory functions of RNASET2, solidifying its potential as a novel therapeutic target for AIT. Our results provide a strategic roadmap for the development of disease-modifying therapies for AIT, including: 1) advancing mechanistic studies to delineate RNASET2’s effects on T-cell and macrophage polarization in thyroid autoimmunity; 2) performing preclinical validation of recombinant RNASET2 or compounds known to modulate its expression (such as genistein) in established experimental models of AIT; and 3) translating these discoveries clinically through biomarker development for patient stratification. By bridging fundamental genetic discovery with functional validation and therapeutic innovation, this work addresses a critical unmet need in AIT management, moving beyond purely symptomatic treatment toward targeted, mechanism-based strategies.

## Data Availability

The original contributions presented in the study are included in the article/**Supplementary Material**. Further inquiries can be directed to the corresponding authors.

## Supplementary material

Supplementary Table 1 MR analysis and sensitivity analysis of the causal effects of eQTL and pQTL on the risk of AIT; **Supplementary Table S2** MR analysis and sensitivity analysis of the causal effects of RNASET2 mQTLs on the risk of AIT; **Supplementary Table S3** Characteristics of significant SNPs with genome-wide associations for eQTL and pQTL of RNASET2 on AIT risk; **Supplementary Table S4** Characteristics of significant SNPs with genome-wide associations for mQTLs of RNASET2 on AIT risk; **Supplementary Figure S1** The stability in the causal associations between eQTL and pQTL of RNASET2 with the risk of AIT; **Supplementary Figure S2** The stability in the causal associations between mQTLs of RNASET2 with the risk of AIT; **Supplementary Figure S3** The Manhattan plot for PheWAS of RNASET2; **Supplementary Figure S4** RNASET2 treatment exhibited no cytotoxic effects on thyrocyte spheroids; **Supplementary Figure S5** Poly(I:C) stimulation mildly upregulated endogenous RNASET2 expression in thyrocyte spheroids; **Supplementary Figure S6** Validation of RNASET2 knockdown efficiency in human thyrocytes; **Supplementary Figure S7** Individual fluorescence channels of **Figure 9a**.

Supplementary Figure 1 Funnel plots (left), scatter plots (middle), and forest plots for MR leave-one-out sensitivity analysis (right) to visualize the stability in the causal associations between eQTL and pQTL of RNASET2 with the risk of AIT in discovery and replication phases.

Supplementary Figure 2 Funnel plots (left), scatter plots (middle), and forest plots for MR leave-one-out sensitivity analysis (right) to visualize the stability in the causal associations between mQTLs of RNASET2 with the risk of AIT in discovery and replication phases.

Supplementary Figure 3 The Manhattan plot for PheWAS of RNASET2.

Supplementary Figure 4 RNASET2 treatment exhibited no cytotoxic effects on thyrocyte spheroids. Spheroids were treated with increasing concentrations of recombinant RNASET2 for 24 hours. Cell viability was assessed using luminescent 3D cell viability assays **(a)**, and viability/cytotoxicity was further evaluated by calcein-AM/PI live/dead staining **(b-d)**. No significant cytotoxic effect was observed across the tested concentration range.

Supplementary Figure 5 Poly(I:C) stimulation mildly upregulated endogenous RNASET2 expression in thyrocyte spheroids. Spheroids were transfected with increasing doses of poly(I:C) (0.05-0.4 µg per spheroid) for 24 hours. **(a)** RNASET2 mRNA levels were quantified by RT–PCR and normalized to GAPDH. **(b)** Secreted RNASET2 protein levels in spheroid supernatants were measured by ELISA. Data represent mean ± SEM. Significance was analyzed by one-way ANOVA followed by Dunnett’s test (*P < 0.05, **P < 0.01, ***P < 0.001, ****P < 0.0001, ns: not significant). Control: non-transfected spheroids; mock: spheroids treated with transfection reagent only.

Supplementary Figure 6 Validation of RNASET2 knockdown efficiency in human thyrocytes. Nthy-ori-31 cells were cultured as monolayers in 6-well plates and transfected with one of three candidate siRNAs targeting human RNASET2 (#1, #2, #3) or the negative control siRNA (siRNA_NC). Forty-eight hours later, cells were stimulated with poly(I:C) (1 µg per well). **(a)** Total RNA was extracted 24-hour post stimulation, and RNASET2 mRNA levels were quantified by RT–PCR and normalized to *GAPDH*. **(b)** Secreted RNASET2 protein in supernatants was quantified by ELISA. Data represent mean ± SEM. Significance was determined by one-way ANOVA followed by Dunnett’s test (****P < 0.0001, ns: not significant). ‘-’: not added; ‘+’: added.

Supplementary Figure 7 Individual fluorescence channels of **Figure 9a**. Thyrocyte spheroids, with or without RNASET2 knockdown, were stimulated with increasing doses of poly(I:C) (0.05-0.4 µg per spheroid) for 24 hours in the absence or presence of 500 ng/ml recombinant RNASET2 treatment. Fluorescence micrographs show viability (Calcein-AM, green) and cell death (PI, red) in spheroids. Control: non-transfected spheroids; siRNA_NC: spheroids transfected with the negative control siRNA; siRNA_RNASET2: RNASET2-knockdown spheroids; siRNA+rRNASET: RNASET2-knockdown spheroids supplemented with recombinant RNASET2 protein.
