# Supplementary material for: Integrative multi-omics Mendelian randomization and functional validation identifies RNASET2 as a novel therapeutic target for autoimmune thyroiditis
Source: Front Endocrinol (Lausanne). 2026 Feb 2;17:1715937. doi: 10.3389/fendo.2026.1715937 (PMC12907157; doi:10.3389/fendo.2026.1715937)

Supplementary Figure S1

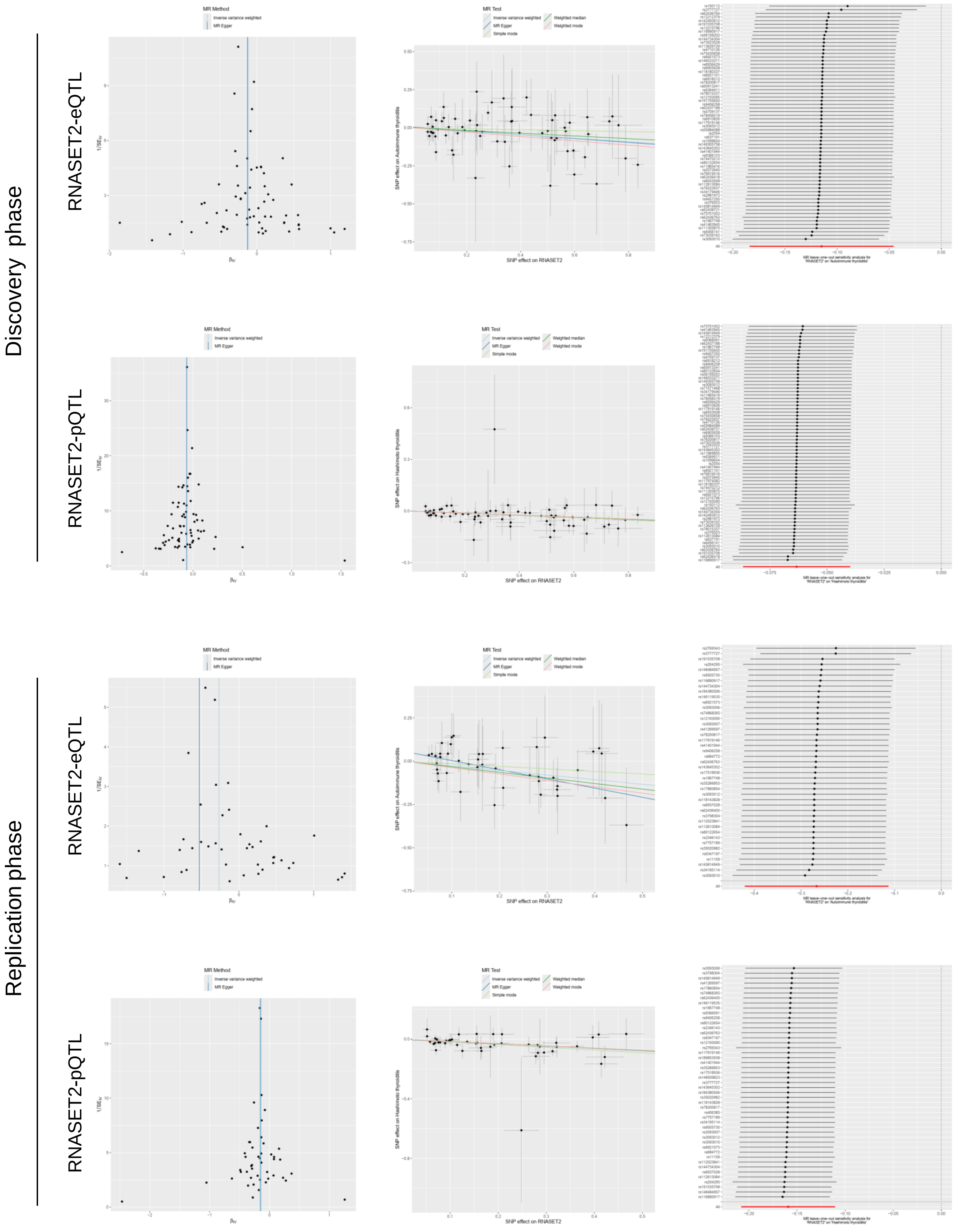

Supplementary Figure S2

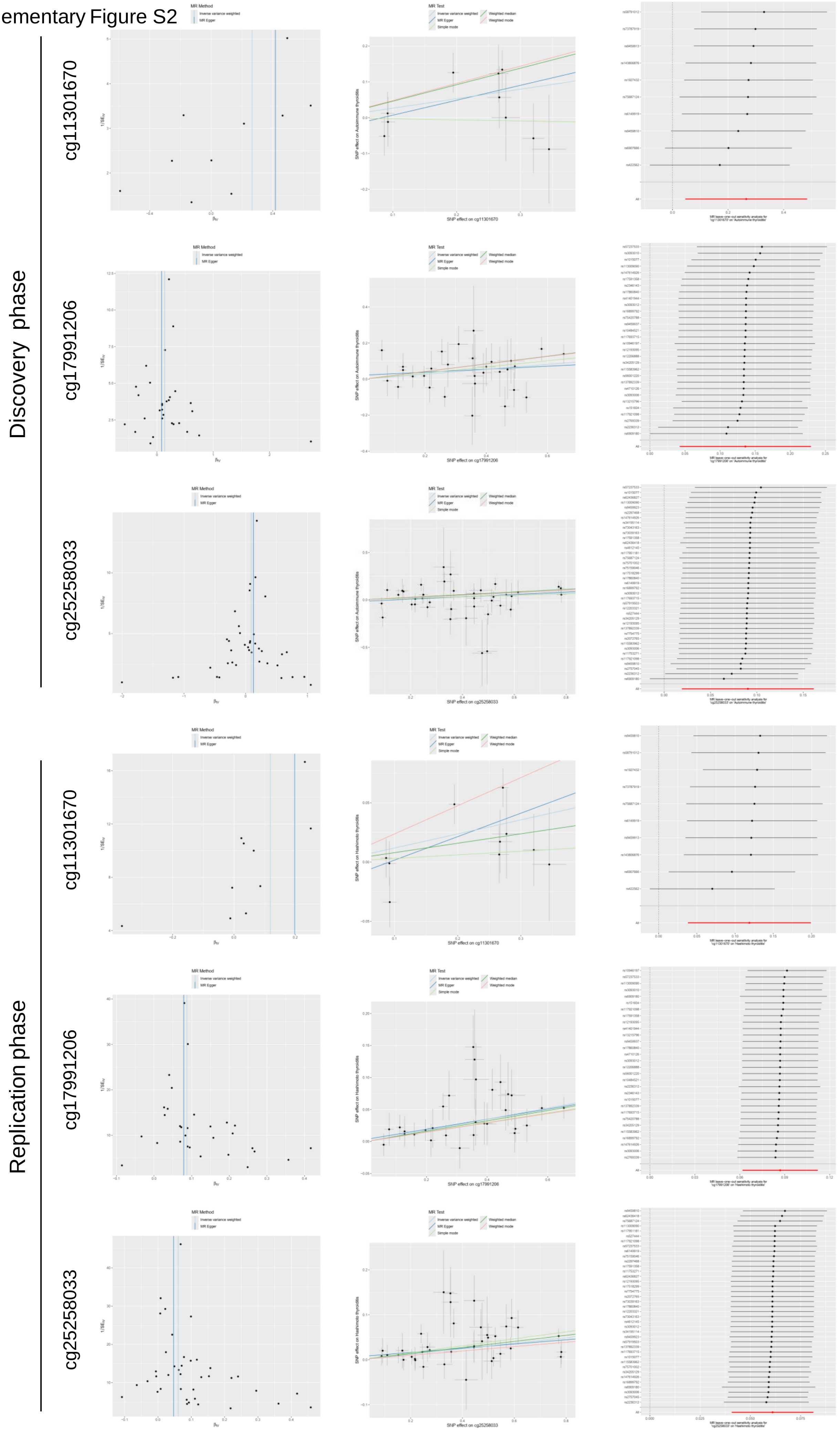

Supplementary Figure S3

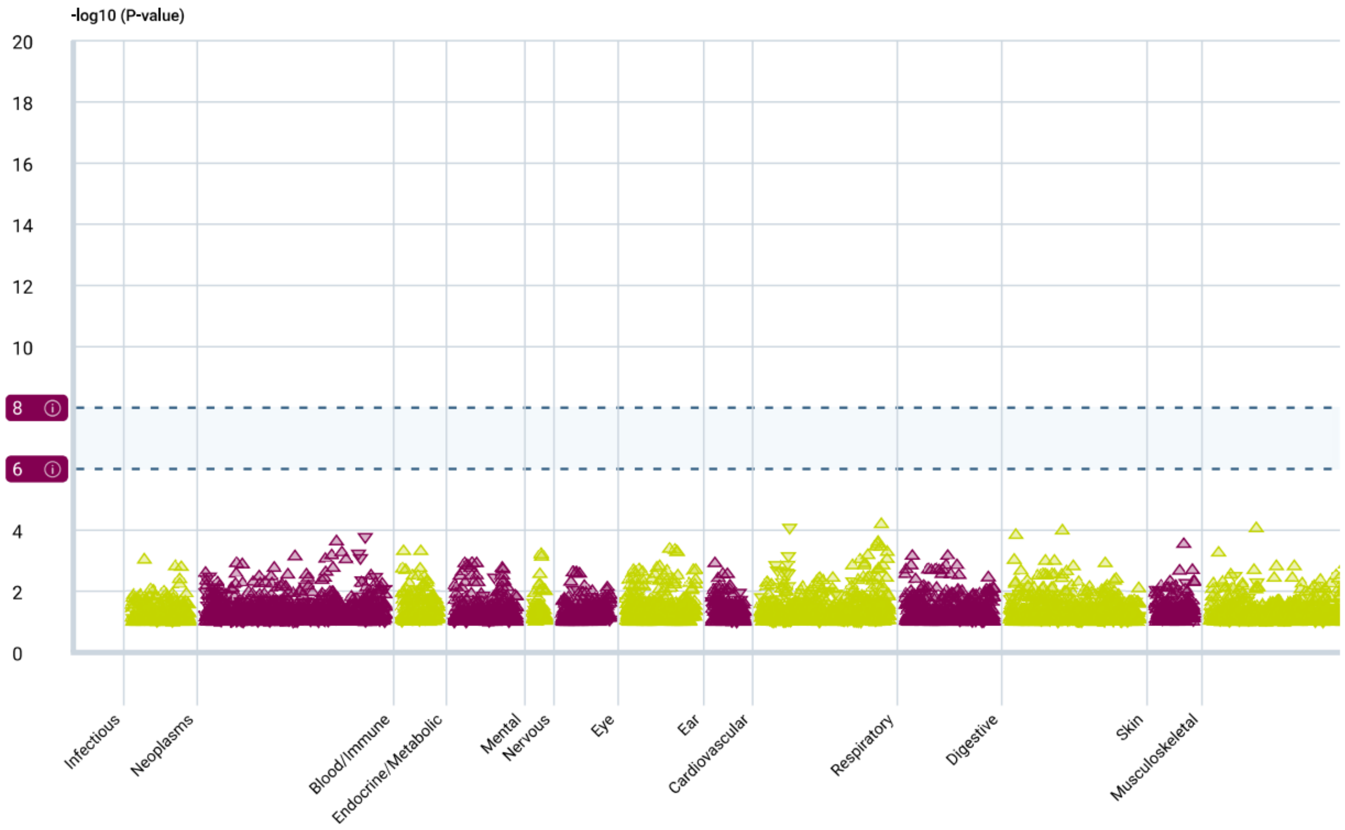

Supplementary Figure S4

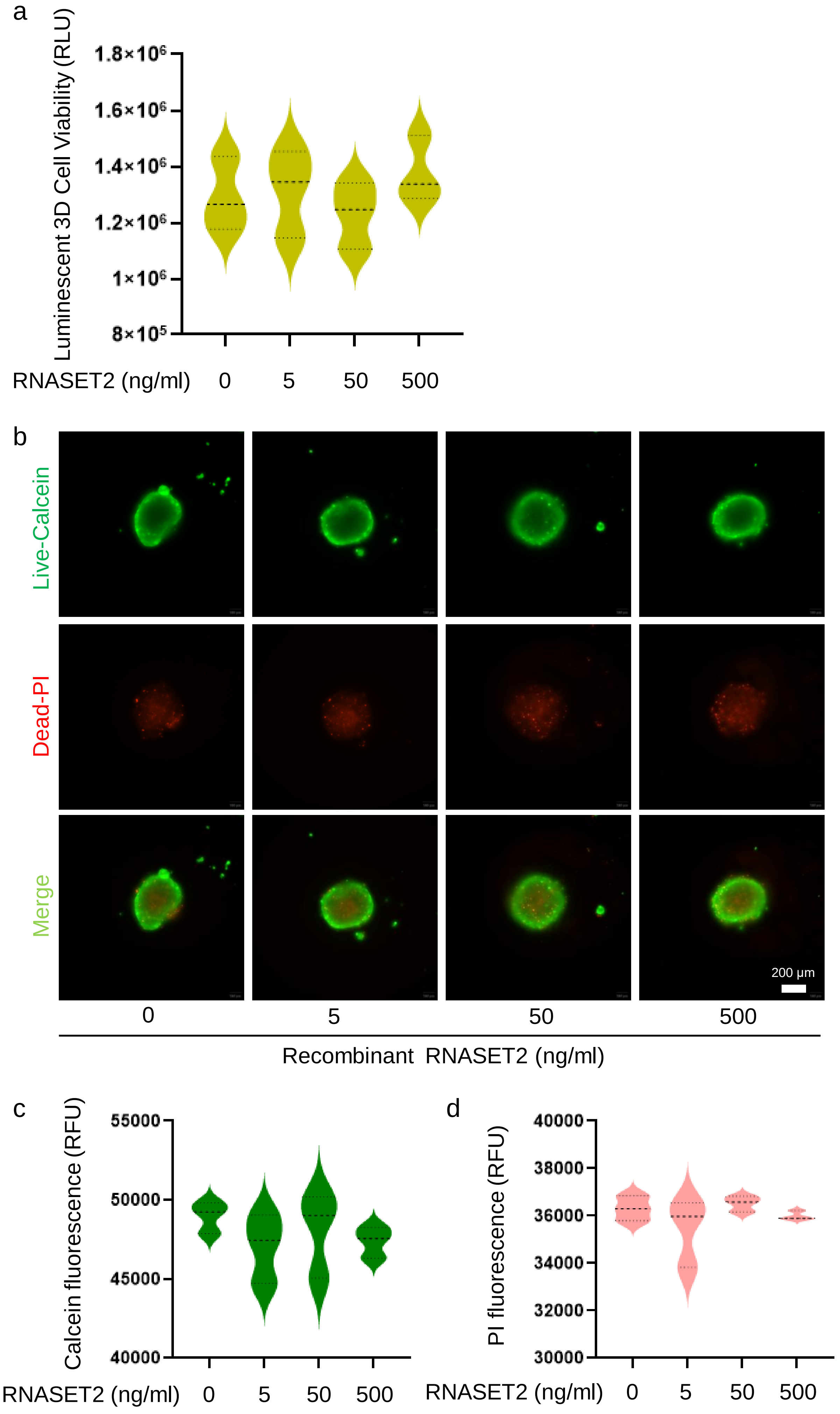

Supplementary Figure S5

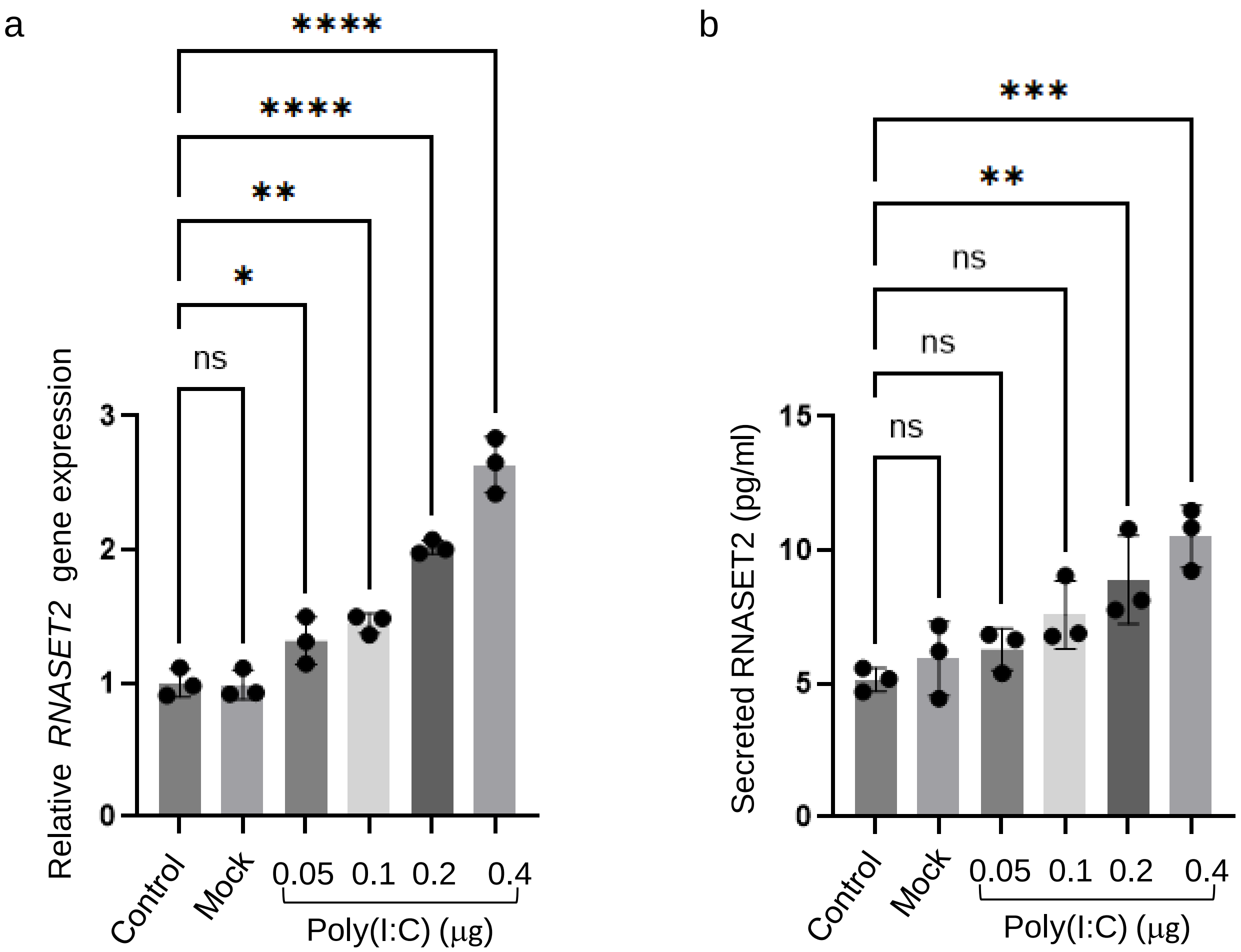

Supplementary Figure S6

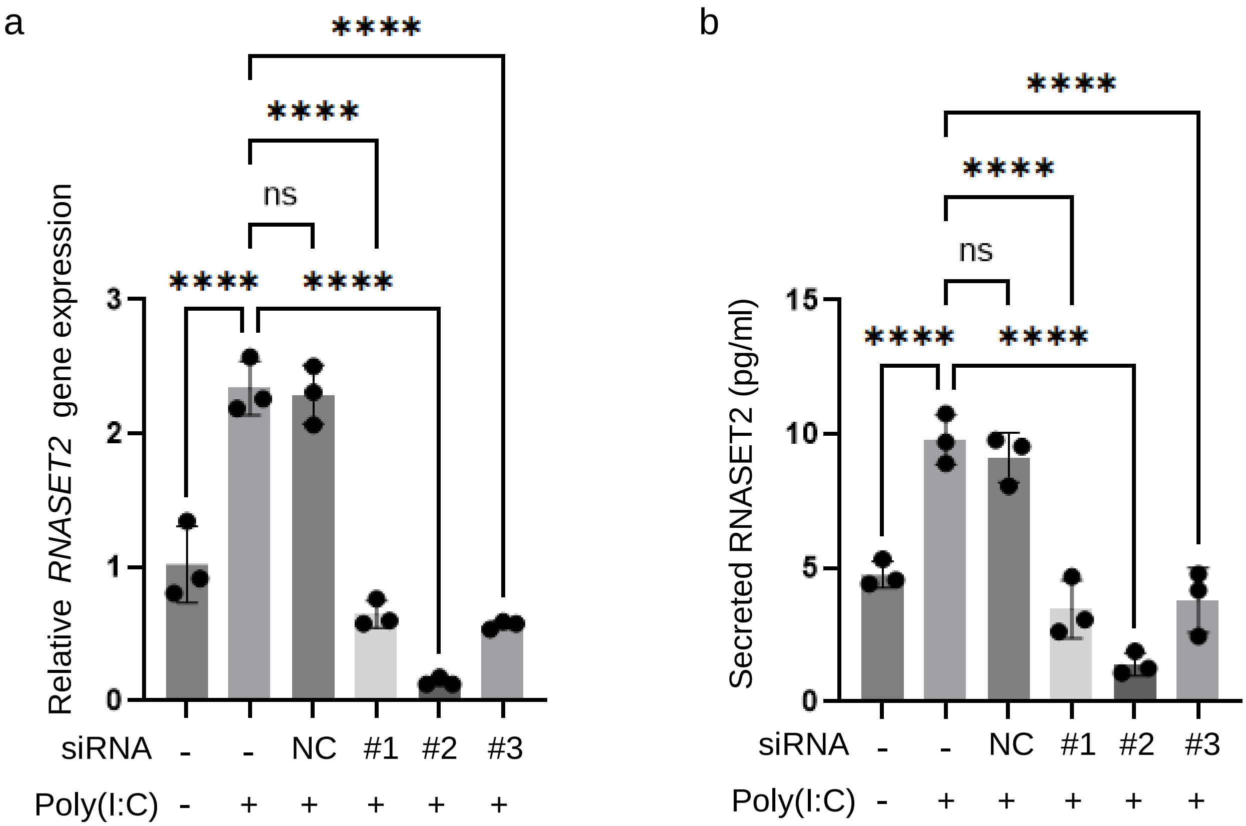

Supplementary Figure S7

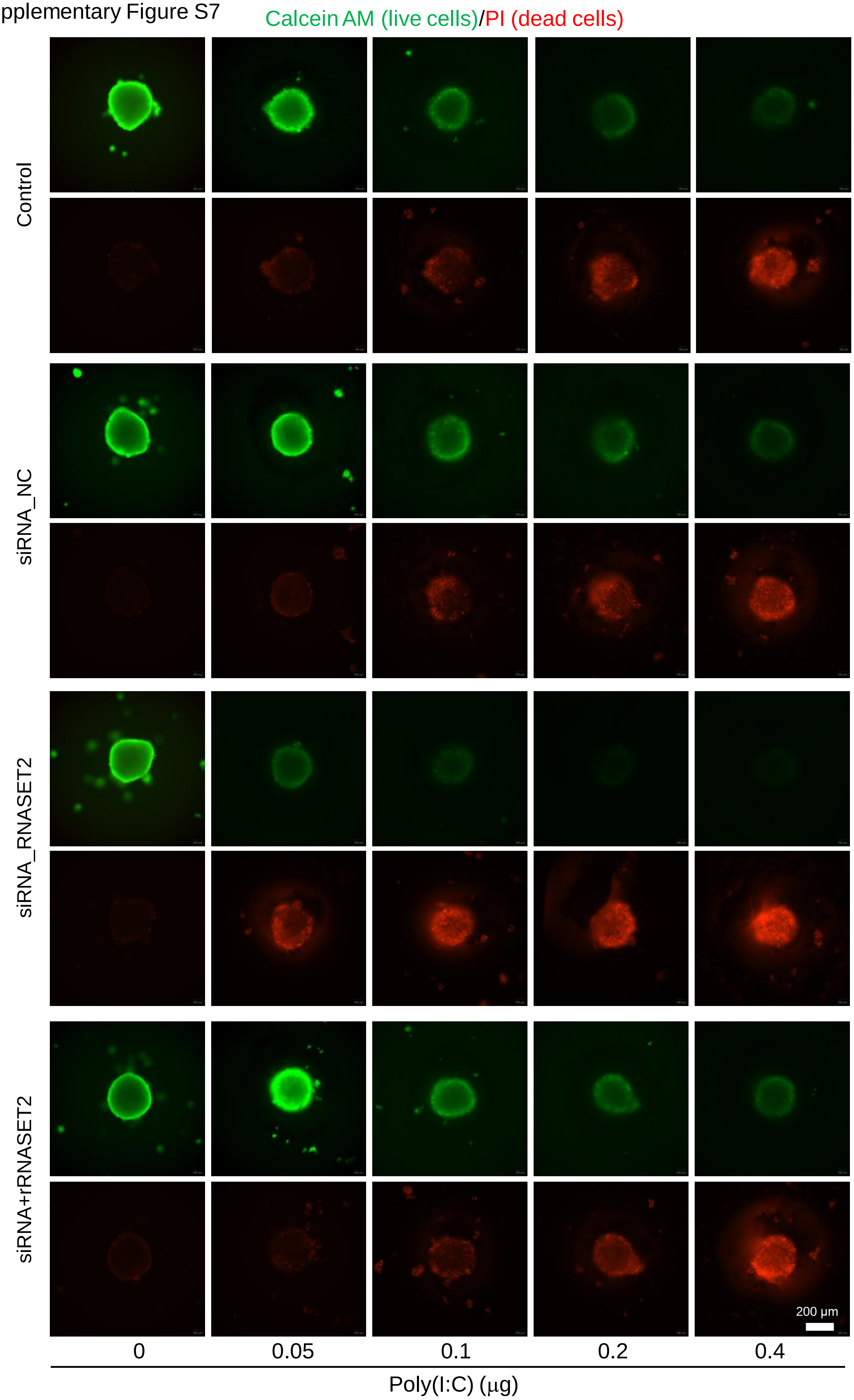

Supplement: Supplementary Figure 1 — Funnel plots (left), scatter plots (middle), and forest plots for MR leave-one-out sensitivity analysis (right) to visualize the stability in the causal associations between eQTL and pQTL of RNASET2 with the risk of AIT in discovery and replication phases. [file DataSheet1.pdf]
