# Supplementary material for: Integrative multi-omics Mendelian randomization and functional validation identifies RNASET2 as a novel therapeutic target for autoimmune thyroiditis
Source: Front Endocrinol (Lausanne). 2026 Feb 2;17:1715937. doi: 10.3389/fendo.2026.1715937 (PMC12907157; doi:10.3389/fendo.2026.1715937)
Supplement: Supplementary Figure 3 — The Manhattan plot for PheWAS of RNASET2. [file DataSheet2.docx]

**STROBE-MR checklist of recommended items to address in reports of Mendelian randomization studies**

| **Item No.** | **Section** | **Checklist item** | **Section name** | **Relevant text from manuscript** |
| --- | --- | --- | --- | --- |
| 1 | **TITLE and ABSTRACT** | **Multi-omics Mendelian randomization identified RNASET2 as a potential therapeutic target for autoimmune thyroiditis** | Title  Abstract | Integrative multi-omics Mendelian randomization and functional validation identifies RNASET2 as a novel therapeutic target for autoimmune thyroiditis  *Objective*: Autoimmune thyroiditis (AIT), a prevalent autoimmune disorder often leading to hypothyroidism, lacks disease-modifying therapies targeting its underlying pathogenesis. Therefore, to develop targeted drugs that directly modulate AIT represents a critical unmet medical need.  Methods: Leveraging integrative genomics, we combined genome-wide association studies (GWAS) with molecular quantitative trait loci (QTL) analyses, including gene expression (eQTL), protein (pQTL), and DNA methylation QTL (mQTL), across two independent AIT cohorts respectively for discovery and replication phases to identify novel therapeutic targets of AIT. We used two-sample bidirectional Mendelian randomization (MR) with sensitivity analyses, and summary-data-based MR (SMR) analysis and heterogeneity in dependent instruments (HEIDI) tests, followed by a comprehensive phenome-wide association study (PheWAS) and computational drug screening and prediction. Guided by these multi-omics findings, we subsequently quantified plasma levels of the top-priority candidate, RNASET2, via ELISA in AIT patients and non-AIT controls. To functionally validate its therapeutic potential, we developed a novel three-dimension (3D) inflammatory thyrocyte spheroid model and evaluated potential therapeutic effects of recombinant RNASET2.  *Results*: Our multi-omics integration consistently nominated RNASET2 as a causal protective factor against AIT. pQTL and eQTL signals linked to RNASET2 were associated with decreased AIT risk, while three mQTLs were correlated with increased risk. PheWAS indicated minimal pleiotropic effects, supporting its therapeutic suitability. Computational drug screening nominated genistein, a soy isoflavone known to upregulate RNASET2 expression, as a repurposing candidate. Importantly, these predictions were empirically supported: we observed significantly elevated plasma RNASET2 levels in AIT patients, a finding potentially reflective of a compensatory anti-inflammatory response to ongoing thyroid tissue damage and inflammation, and demonstrated that recombinant RNASET2 effectively mitigated inflammation and apoptosis in the thyrocyte spheroid model, confirming its functional protective role.  *Conclusions*: Integrating large-scale genomic analyses with functional validation, our study identifies RNASET2 augmentation as a promising therapeutic strategy for AIT, providing a translational bridge from genetic discovery to potential clinical application. |
|  | **INTRODUCTION** |  | Introduction |  |
| 2 | **Background** | Explain the scientific background and rationale for the reported study. What is the exposure? Is a potential causal relationship between exposure and outcome plausible? Justify why MR is a helpful method to address the study question | Introduction | “Effective drug development relies on the accurate identification of therapeutic targets and rigorous validation of their disease-modifying on a disease. However, conventional drug discovery approaches face significant challenges including high costs, lengthy timelines, and substantial failure rates. To overcome these limitations, innovative strategies integrating genomic data are essential for accelerating the process of therapeutic target identification [2]. In recent years, the combined analysis of genome-wide association studies (GWAS) with molecular quantitative trait loci (QTL) data, such as expression QTLs (eQTLs), DNA methylation QTLs (mQTLs), and protein QTLs (pQTLs), provides a powerful framework for establishing causal relationships between genetic variants and disease-associated molecules [3,4]. This integrative approach enhances the discovery of biologically relevant drug targets while improving the efficiency of the drug development pipeline.  Mendelian randomization (MR) analysis serves as a powerful analytical approach to assess causal relationships between exposures and outcomes by leveraging genetic variants as instrumental variables (IVs), effectively mimicking a randomized controlled trial using observational genetic data [5]. This method provides a robust alternative to traditional drug testing by circumventing confounding factors and reverse causation inherent in conventional observational studies [5]. Notably, MR analysis has been successfully applied to discover drug targets in several autoimmune disorders [6-9], offering novel insights into their pathogenesis and potential therapeutic targets.” |
| 3 | **Objectives** | State specific objectives clearly, including pre-specified causal hypotheses (if any). State that MR is a method that, under specific assumptions, intends to estimate causal effects | Introduction | “In this study, we aimed to identify novel therapeutic targets for AIT by selecting instrumental variables associated with eQTLs, mQTLs, and pQTLs to investigate their causal effects on gene expression, epigenetic modifications, and protein levels in AIT. To enhance the robustness of our findings, we validated the results using two independent GWAS datasets of AIT and employed a two-sample MR analysis as our primary analytical approach, complemented with summary-data-based MR (SMR) analysis and heterogeneity in dependent instruments (HEIDI) tests.” |
|  | **METHODS** |  | Methods |  |
|  | **Study design and data sources** | Present key elements of the study design early in the article. Consider including a table listing sources of data for all phases of the study. For each data source contributing to the analysis, describe the following: |  |  |
|  | a) | Setting: Describe the study design and the underlying population, if possible. Describe the setting, locations, and relevant dates, including periods of recruitment, exposure, follow-up, and data collection, when available. | *Study design* | “Our large-data analysis (**Figure 1**) integrated GWAS data for molecular QTLs (cis-eQTLs, pQTLs, and mQTLs) with two independent AIT GWAS datasets (discovery and replication cohorts) to investigate causal relationships. After rigorous IV selection, we conducted two-sample MR analyses to assess causal effects of gene expression and protein levels on AIT risk, with sensitivity analyses for reverse causation, horizontal pleiotropy, and heterogeneity, and with cross-validation of directional effects between cohorts. Significant findings underwent additional validation through SMR and HEIDI test at the gene expression level, followed by mQTL-specific MR analysis to evaluate DNA methylation effects of the identified gene on AIT. Finally, we performed phenome-wide association study (PheWAS) and computational drug prediction for the identified target, with detailed methodological specifications provided in subsequent sections.” |
|  | b) | Participants: Give the eligibility criteria, and the sources and methods of selection of participants. Report the sample size, and whether any power or sample size calculations were carried out prior to the main analysis | *Data sources* | “We obtained GWAS data for AIT from two independent sources: 1) the FinnGen consortium's latest release (R12), comprising 688 cases and 424,208 controls (accession: finngen_R12_E4_THYROIDITAUTOIM), and 2) the IEU Open GWAS project with 15,654 cases and 379,986 controls (accession: ebi-a-GCST90018855) [11]. For molecular QTLs, we utilized: (i) cis-eQTL data from the eQTLGen consortium (https://eqtlgen.org/) (31,684 blood samples; 16,989 genes) [12], (ii) pQTL data from UKB-PPP (http://ukb-ppp.gwas.eu) (54,219 participants; 2,923 plasma proteins) [13], and (iii) mQTL data from GoDMC (http://mqtldb.godmc.org.uk/) (27,750 blood samples; 420,509 DNA methylation sites) [14]. Additionally, we referred druggable gene information from established pharmacogenomic resources [15] to facilitate therapeutic target identification. All data sources are publicly available through their respective consortium portals.” |
|  | c) | Describe measurement, quality control and selection of genetic variants | *Selection of IVs* | “To ensure valid causal inference using genetic variants as IVs, we adhered to three core MR assumptions: 1) strong association between genetic variants and exposure (relevance assumption), 2) absence of confounding between genetic variants and outcome (independence assumption), and 3) exclusion of pleiotropic pathways not mediated by the exposure (exclusion restriction assumption). Our IV selection protocol incorporated the following stringent criteria: First, we identified potential IVs through genome-wide screening of molecular QTLs, applying locus-specific significance thresholds (P < 5×10^-8^ for eQTLs; P < 1×10^-5^ for pQTLs/mQTLs). Second, we performed linkage disequilibrium (LD)-based clumping using European samples from the 1000 Genomes Project as reference, retaining only independent variants (R^2^ < 0.001 within 10,000 kb windows) with the strongest association signals [16]. Third, we evaluated instrument strength using the F-statistic [(N-K-1)/K × R^2^/(1-R^2^)], excluding variants with F < 10 to mitigate weak instrument bias [17]. Finally, we systematically removed confounding variants: those in the MHC region (Chr6:26-34 Mb) [18], to ensure the validity of our causal estimates.” |
|  | d) | For each exposure, outcome, and other relevant variables, describe methods of assessment and diagnostic criteria for diseases | *Data sources* | Details could be found in each original studies cited in *Data source* section. |
|  | e) | Provide details of ethics committee approval and participant informed consent, if relevant | *Ethical approval* | “The data used in our analysis were publicly available and had been approved by the respective institutional review boards of the original studies.” |
| 5 | **Assumptions** | Explicitly state the three core IV assumptions for the main analysis (relevance, independence and exclusion restriction) as well assumptions for any additional or sensitivity analysis | *Selection of IVs* | “To ensure valid causal inference using genetic variants as IVs, we adhered to three core MR assumptions: 1) strong association between genetic variants and exposure (relevance assumption), 2) absence of confounding between genetic variants and outcome (independence assumption), and 3) exclusion of pleiotropic pathways not mediated by the exposure (exclusion restriction assumption).” |
| 6 | **Statistical methods: main analysis** | Describe statistical methods and statistics used |  |  |
|  | a) | Describe how quantitative variables were handled in the analyses (i.e., scale, units, model) | *Data sources* | Quantitative variables in the analyses can be referred from each original studies cited in *Data source* section. |
|  | b) | Describe how genetic variants were handled in the analyses and, if applicable, how their weights were selected | *Selection of IVs* | “To ensure valid causal inference using genetic variants as IVs, we adhered to three core MR assumptions: 1) strong association between genetic variants and exposure (relevance assumption), 2) absence of confounding between genetic variants and outcome (independence assumption), and 3) exclusion of pleiotropic pathways not mediated by the exposure (exclusion restriction assumption). Our IV selection protocol incorporated the following stringent criteria: First, we identified potential IVs through genome-wide screening of molecular QTLs, applying locus-specific significance thresholds (P < 5×10^-8^ for eQTLs; P < 1×10^-5^ for pQTLs/mQTLs). Second, we performed linkage disequilibrium (LD)-based clumping using European samples from the 1000 Genomes Project as reference, retaining only independent variants (R^2^ < 0.001 within 10,000 kb windows) with the strongest association signals [16]. Third, we evaluated instrument strength using the F-statistic [(N-K-1)/K × R^2^/(1-R^2^)], excluding variants with F < 10 to mitigate weak instrument bias [17]. Finally, we systematically removed confounding variants: those in the MHC region (Chr6:26-34 Mb) [18], to ensure the validity of our causal estimates.” |
|  | c) | Describe the MR estimator (e.g. two-stage least squares, Wald ratio) and related statistics. Detail the included covariates and, in case of two-sample MR, whether the same covariate set was used for adjustment in the two samples | *Two-sample MR analysis* | “We conducted a comprehensive two-sample MR analysis to investigate potential causal relationships between molecular QTLs and AIT. Our primary analytical approach utilized the inverse variance weighted (IVW) method, which combines Wald ratio estimates from individual SNPs through meta-analysis to provide robust causal effect estimates [19]. The analysis protocol consisted of three key steps: (1) harmonization of SNP alleles across datasets to ensure proper matching, (2) calculation of Wald ratio estimates for each individual SNP, and (3) meta-analysis integration using the IVW approach. This method offers enhanced precision by leveraging multiple genetic variants simultaneously while maintaining unbiased estimates in the absence of horizontal pleiotropy. Effect sizes were expressed as odds ratios (ORs) derived from exponentiated β coefficients, accompanied by 95% confidence intervals (CIs), with statistical significance defined as P < 0.05.  To ensure the robustness of our findings, we implemented multiple validation strategies: (1) sensitivity analyses using MR-Egger regression (which corrects for pleiotropic bias through linear regression modeling) [20], weighted median estimator (providing reliable estimates under sample bias) [21], simple mode and weighted mode approaches (reducing confounding through randomization techniques) [22]; (2) stringent exclusion of potential reverse causation by retaining only SNPs with greater variance explained in exposure than outcome; and (3) preservation of data integrity by abstaining from missing value imputation. All analyses were performed using the "TwoSampleMR" package within the R 4.3.2 statistical environment (available at https://cran.r-project.org/bin/windows/base/).” |
|  | d) | Explain how missing data were addressed | *Two-sample MR analysis* | “…and (3) preservation of data integrity by abstaining from missing value imputation.” |
|  | e) | If applicable, indicate how multiple testing was addressed | *Two-sample MR analysis*  *SMR analysis and HEIDI test* | “To ensure the robustness of our findings, we implemented multiple validation strategies: (1) sensitivity analyses using MR-Egger regression (which corrects for pleiotropic bias through linear regression modeling) [20], weighted median estimator (providing reliable estimates under sample bias) [21], simple mode and weighted mode approaches (reducing confounding through randomization techniques) [22]; (2) stringent exclusion of potential reverse causation by retaining only SNPs with greater variance explained in exposure than outcome; and (3) preservation of data integrity by abstaining from missing value imputation.”  “The SMR and HEIDI approach offers a distinct advantage over most other integrative methods for analyzing GWAS and eQTL data by enabling differentiation between pleiotropic and linkage models [25]. We employed SMR analysis as a supplementary approach to validate causal relationships between AIT and gene expression. The HEIDI test was specifically applied to rule out the possibility of genetic linkage [26]. Following established criteria, we defined significant SMR associations as those with P < 0.05, while HEIDI results with P > 0.05 indicated associations mediated by shared genetic variants rather than linkage. These additional analyses strengthened the evidence for the causal relationships identified through our primary MR analysis.” |
| 7 | **Assessment of assumptions** | Describe any methods or prior knowledge used to assess the assumptions or justify their validity | *Two-sample MR analysis* | “We conducted a comprehensive two-sample MR analysis to investigate potential causal relationships between molecular QTLs and AIT. Our primary analytical approach utilized the inverse variance weighted (IVW) method, which combines Wald ratio estimates from individual SNPs through meta-analysis to provide robust causal effect estimates [19]. The analysis protocol consisted of three key steps: (1) harmonization of SNP alleles across datasets to ensure proper matching, (2) calculation of Wald ratio estimates for each individual SNP, and (3) meta-analysis integration using the IVW approach. This method offers enhanced precision by leveraging multiple genetic variants simultaneously while maintaining unbiased estimates in the absence of horizontal pleiotropy. Effect sizes were expressed as odds ratios (ORs) derived from exponentiated β coefficients, accompanied by 95% confidence intervals (CIs), with statistical significance defined as P < 0.05.  To ensure the robustness of our findings, we implemented multiple validation strategies: (1) sensitivity analyses using MR-Egger regression (which corrects for pleiotropic bias through linear regression modeling) [20], weighted median estimator (providing reliable estimates under sample bias) [21], simple mode and weighted mode approaches (reducing confounding through randomization techniques) [22]; (2) stringent exclusion of potential reverse causation by retaining only SNPs with greater variance explained in exposure than outcome; and (3) preservation of data integrity by abstaining from missing value imputation. All analyses were performed using the "TwoSampleMR" package within the R 4.3.2 statistical environment (available at https://cran.r-project.org/bin/windows/base/).” |
| 8 | **Sensitivity analyses and additional analyses** | Describe any sensitivity analyses or additional analyses performed (e.g. comparison of effect estimates from different approaches, independent replication, bias analytic techniques, validation of instruments, simulations) | *Sensitivity analysis* | “Heterogeneity among the IVs was assessed using Cochran's Q test, calculated within the IVW framework. Cochran's Q test is a widely used method to evaluate heterogeneity across different IVs in a study [23]. The P-value obtained from this test is critical in determining the presence or absence of significant heterogeneity. P < 0.05 indicates significant heterogeneity among the IVs. To address potential horizontal pleiotropy, the MR-Egger intercept test was employed [20]. The significance of the intercept term in the MR-Egger regression suggests the presence of horizontal pleiotropy. Additionally, the MR pleiotropy residual sum and outlier (MR-PRESSO) method was applied to identify and exclude outliers with horizontal pleiotropic effects that could significantly bias the MR estimates [24]. To visually assess the robustness of the results, scatter plots and funnel plots were generated. Scatter plots confirmed that the findings were not influenced by outliers, while funnel plots demonstrated the consistency and absence of heterogeneity in the correlations. Finally, the "leave-one-out" method was used to evaluate the impact of individual SNPs on the MR estimates. This approach involved iteratively excluding one SNP at a time to determine whether the removal of any single SNP substantially altered the causal estimates, thereby ensuring the stability and reliability of the results.” |
| 9 | **Software and pre-registration** |  |  |  |
|  | a) | Name statistical software and package(s), including version and settings used | *Two-sample MR analysis* | “All analyses were performed using the "TwoSampleMR" package within the R 4.3.2 statistical environment (available at https://cran.r-project.org/bin/windows/base/).” |
|  | b) | State whether the study protocol and details were pre-registered (as well as when and where) | - | Not applicable. |
|  | **RESULTS** |  |  |  |
| 10 | **Descriptive data** |  |  |  |
|  | a) | Report the numbers of individuals at each stage of included studies and reasons for exclusion. Consider use of a flow diagram | *Data sources* | Details could be found in each original studies cited in *Data source* section. |
|  | b) | Report summary statistics for phenotypic exposure(s), outcome(s), and other relevant variables (e.g. means, SDs, proportions) | *Data sources* | Details could be found in each original studies cited in *Data source* section. |
|  | c) | If the data sources include meta-analyses of previous studies, provide the assessments of heterogeneity across these studies | Results | Analysis results for heterogeneity were all included in **Supplementary Tables S1-S2**. |
|  | d) | For two-sampleMR:  i.  Provide justification of the similarity of the genetic variant-exposure associations between the exposure and outcome samples  ii.  Provide information on the number of individuals who overlap between the exposure and outcome studies | Results | Characteristics of significant SNPs with genome-wide associations for eQTL, pQTL, and mQTL of RNASET2 with AIT risk were summarized in **Supplementary Table S3-S4**. |
| 11 | **Main results** |  |  |  |
|  | a) | Report the associations between genetic variant and exposure, and between genetic variant and outcome, preferably on an interpretable scale | Results | Causal associations between eQTL, pQTL, and mQTL of RNASET2 with AIT risk: **Figure 3-5**, and **Supplementary Table S1-S2.** |
|  | b) | Report MR estimates of the relationship between exposure and outcome, and the measures of uncertainty from the MR analysis, on an interpretable scale, such as odds ratio or relative risk per SD difference | Results | Information including OR, 95% CI, and p values for eQTL, pQTL, and mQTL of RNASET2 with AIT risk incorporated into **Figure 3-5**, and **Supplementary Table S1-S2.** |
|  | c) | If relevant, consider translating estimates of relative risk into absolute risk for a meaningful time period |  | Not applicable. |
|  | d) | Consider plots to visualize results (e.g. forest plot, scatter plot of associations between genetic variants and outcome versus between genetic variants and exposure) | Results | Forest plots: **Figures 3, 5**.  Scatter plots: **Supplementary Figures S1-S2**.  Funnel plots: **Supplementary Figures S1-S2**.  “Leave-one-out sensitivity” plots: **Supplementary Figures S1-S2**.. |
| 12 | **Assessment of assumptions** |  |  |  |
|  | a) | Report the assessment of the validity of the assumptions | Results | Assessments of the validity of the assumptions employed five methods, and were included in **Figures 3, 5** and **Supplementary Tables S1-S2**. |
|  | b) | Report any additional statistics (e.g., assessments of heterogeneity across genetic variants, such as *I^2^*, Q statistic or E-value) | Results | Assessments for heterogeneity were included in **Supplementary Tables S1-2**. |
| 13 | **Sensitivity analyses and additional analyses** |  |  |  |
|  | a) | Report any sensitivity analyses to assess the robustness of the main results to violations of the assumptions | Results | Sensitivity analyses for horizontal pleiotropy and heterogeneity were included in **Supplementary** **Tables S1**-**S2** and **Table 1**. |
|  | b) | Report results from other sensitivity analyses or additional analyses | Results | Leave-one-out analyses were all included in **Supplementary Figures** **S1-S2**. |
|  | c) | Report any assessment of direction of causal relationship (e.g., bidirectional MR) | Results | Results of reverse causal associations between eQTL, pQTL, and mQTL of RNASET2 with AIT risk: **Figure 3-5**, and **Supplementary Table S1-S2.** |
|  | d) | When relevant, report and compare with estimates from non-MR analyses |  | Not applicable. |
|  | e) | Consider additional plots to visualize results (e.g., leave-one-out analyses) | Results | Additional plots were all included in **Supplementary Figures** **S1-S2**. |
|  | **DISCUSSION** |  |  |  |
| 14 | **Key results** | Summarize key results with reference to study objectives | Discussion | “By integrating GWAS data analyzing cis-eQTLs, pQTLs, and mQTLs with two independent AIT GWAS datasets, we identified RNASET2 as a novel causal therapeutic target for AIT.” |
| 15 | **Limitations** | Discuss limitations of the study, taking into account the validity of the IV assumptions, other sources of potential bias, and imprecision. Discuss both direction and magnitude of any potential bias and any efforts to address them | Discussion | “While our study provides solid evidence for RNASET2 as a therapeutic target in AIT, several limitations should be acknowledged. First, the large-scale data used in this study were derived from European populations, which may limit the generalizability of our findings to other ethnic groups. Second, our analyses primarily relied on blood-derived QTL data, which may not fully capture tissue-specific regulatory mechanisms in the thyroid gland. Third, the observational nature of GWAS data means that despite rigorous MR approaches, residual confounding cannot be completely ruled out. Fourth, the sample sizes of both the AIT patient and non-AIT control groups were relatively small, which may limit the statistical power and generalizability of the findings. Therefore, further validation in larger, independent cohorts is necessary to confirm these results. Moreover, *in vivo* studies are essential to substantiate the protective effects of RNASET2 observed in the thyrocyte spheroid model and to assess its physiological relevance in a whole-organism context. Also, given the potential pleiotropic functions of RNASET2 in other biological pathways, future preclinical studies should carefully evaluate its broader systemic effects to fully understand its efficacy and safety profile. These limitations highlight important considerations for translating our findings into clinical applications.” |
| 16 | **Interpretation** |  |  |  |
|  | a) | Meaning: Give a cautious overall interpretation of results in the context of their limitations and in comparison with other studies | Conclusion | “Through integrative genomic and epigenomic analyses, we have established RNASET2 as a causal protective factor in AIT.” |
|  | b) | Mechanism: Discuss underlying biological mechanisms that could drive a potential causal relationship between the investigated exposure and the outcome, and whether the gene-environment equivalence assumption is reasonable. Use causal language carefully, clarifying that IV estimates may provide causal effects only under certain assumptions | Discussion | We discussed the underlying biological mechanisms that might drive a potential causal relationship between RNASET2 and AIT in the discussion section: “The human *RNASET2* gene encodes ribonuclease T2 (RNAse T2), the sole characterized member of the Rh/T2/S family of acid hydrolases in humans [33]……Thus, RNASET2 expression is regulated by multilevel transcriptional and post-transcriptional regulatory mechanism complexity modulating, ultimately driving divergent cellular phenotypes [44].” |
|  | c) | Clinical relevance: Discuss whether the results have clinical or public policy relevance, and to what extent they inform effect sizes of possible interventions | Conclusion | “Our results provide a strategic roadmap for the development of disease-modifying therapies for AIT, including: 1) advancing mechanistic studies to delineate RNASET2's effects on T-cell and macrophage polarization in thyroid autoimmunity; 2) performing preclinical validation of recombinant RNASET2 or compounds known to modulate its expression (such as genistein) in established experimental models of AIT; and 3) translating these discoveries clinically through biomarker development for patient stratification. By bridging fundamental genetic discovery with functional validation and therapeutic innovation, this work addresses a critical unmet need in AIT management, moving beyond purely symptomatic treatment toward targeted, mechanism-based strategies.” |
| 17 | **Generalizability** | Discuss the generalizability of the study results (a) to other populations, (b) across other exposure periods/timings, and (c) across other levels of exposure | Discussion | Study results were deemed generalizable at European level, and discussed as such: “First, the data used in this study were derived from European populations, which may limit the generalizability of our findings to other ethnic groups.” |
|  | **OTHER INFORMATION** |  |  |  |
| 18 | **Funding** | Describe sources of funding and the role of funders in the present study and, if applicable, sources of funding for the databases and original study or studies on which the present study is based | Funding | This work was supported by the National Natural Science Foundation of China (#81900712) and Project of Institute of Chinese Medicine, Nanjing University (#ICM2024013). |
| 19 | **Data and data sharing** | Provide the data used to perform all analyses or report where and how the data can be accessed, and reference these sources in the article. Provide the statistical code needed to reproduce the results in the article, or report whether the code is publicly accessible and if so, where | Data availability statement | All datasets generated during the current study are included in the article/supplementary materials. |
| 20 | **Conflicts of Interest** | All authors should declare all potential conflicts of interest | Declaration of interest | The authors declare no conflicts of interest. |

This checklist is copyrighted by the Equator Network under the Creative Commons Attribution 3.0 Unported (CC BY 3.0) license.
